# An inducer-independent, single-plasmid CRISPR-Cas9 system for genome editing in *Bacillus* species

**DOI:** 10.1007/s00253-026-14030-6

**Published:** 2026-09-11

**Authors:** Maximilian Hilkmann, Norma Welsch, Max Fabian Felle, Thomas Schweder, Mathis Appelbaum

**Affiliations:** 1 Pharmaceutical Biotechnology, Institute of Pharmacy, University of Greifswald, 17487 Greifswald, Germany; 2 Institute of Marine Biotechnology, 17489 Greifswald, Germany; 3 BASF SE, 67063 Ludwigshafen Am Rhein, Germany

**Keywords:** *Bacillus*, CRISPR-Cas, Genome editing, Strain optimization, Promoter

## Abstract

**Abstract:**

Advances in molecular biology tools are essential for streamlining and accelerating genetic engineering of cells across industrial and academic applications. While CRISPR-Cas improves genome editing efficiency, current systems have limitations and are often host specific, which restricts their versatility. This study describes a versatile CRISPR-Cas9 system for genome editing in industrially relevant *Bacillus* species. By adapting the well-established pJOE8999 vector-based CRISPR-Cas9 genome editing system, we constructed an inducer-independent, broad-host-range genome editing system. It maintains the benefits of low toxicity to the target cell and the cloning host as well as the ease to use of a single-plasmid CRISPR-Cas9 system. We utilized the constitutive Sigma70-type promoter from the conserved *veg* gene of *Bacillus*, to develop and test the suitability of promoter variants of different strengths for Cas9 expression. Successful gene deletions in three different *Bacillus* species demonstrated the versatility of the modified system for this industrially important genus. This was further confirmed by the integration of a reporter gene fusion and the introduction of a single point mutation in the genome of *Bacillus licheniformis*. This one-step CRISPR-based transformation protocol developed in this study enables fast genome editing workflows with minimal hands-on time.

**Key points:**

• *Editing and screening of promoter variants for balanced Cas9 expression in Bacillus.*

• *Development of a versatile inducer-independent, single-plasmid CRISPR-Cas-based system.*

• *Verification of the modified CRISPR-based system for genome editing in different Bacilli.*

**Supplementary Information:**

The online version contains supplementary material available at https://doi.org/10.1007/s00253-026-14030-6.

## Introduction

Clustered regularly interspaced short palindromic repeats (CRISPR) and CRISPR associated (Cas) genes encode an adaptive immune system against exogenic elements in archaea and bacteria (Barrangou et al. 2007; Barrangou and Marraffini 2014). The specificity, simplicity and efficiency of CRISPR-Cas in targeting and cleaving nucleic acid strands led to its development as a genome editing tool with unprecedented success, accelerating genome editing in a plethora of species and research fields in academia and industry (Jinek et al. 2012, 2013; Jiang et al. 2013; Hsu et al. 2014). Despite the emphasized simplicity of CRISPR-Cas systems, establishing highly efficient genome editing systems requires proper design and often debottlenecking of individual steps along the editing workflow. This comprises cloning, transformation, transcriptional control of endonuclease expression, toxicity to the target and cloning host as well as curing of the CRISPR-Cas system from the host cell.

*Bacillus* species are major workhorses in industrial biotechnology for the production of enzymes, vitamins and flavor agents among others (Hohmann et al. 2017; Acevedo-Rocha et al. 2019; Appelbaum and Schweder 2021). Moreover, *Bacillus subtilis* serves as the model organism for cellular differentiation, sporulation and Gram-positive bacteria in general and represents one of the best characterized species (Errington and Aart 2020; Appelbaum and Schweder 2021; Pedreira et al. 2022). Various type II CRISPR-Cas9 systems were developed for genome editing of *Bacillus* strains, including *B. subtilis*, *B. licheniformis* and the thermophilic *B. smithii.* The described strategies for delivering the CRISPR-Cas components Cas9, CRISPR RNA (crRNA) and trans-activating crRNA (tracrRNA) or a chimeric single guide RNA (sgRNA) and the homology-directed repair (HDR) template comprise single and dual-plasmid approaches as well as chromosomal integration of the Cas9 expression cassette and optionally the guide RNA. Among these strategies, the pJOE8999 plasmid by Altenbuchner (2016) is one of the earliest and most established CRISPR-Cas9 systems for *B. subtilis* (Altenbuchner 2016; Schilling et al. 2018; Toymentseva and Altenbuchner 2019; Ferrando et al. 2023). In addition, it fulfills most criteria for a simple and efficient CRISPR-Cas9 system from cloning to curing. The pJOE8999 system uses an pUC18 *Escherichia coli*–pE194ts *Bacillus* shuttle-vector single-plasmid approach. This requires only one transformation event to establish a functional genome editing system making it suitable for modification of non-model *Bacillus* strains, which often have low transformation rates (Zhang et al. 2016; Li et al. 2018; Zhou et al. 2019; Schultenkämper et al. 2019). Curing of the plasmid generates a clean strain background. Both factors determine speed and simplicity of the editing protocol and allow for a large degree of parallelization in strain construction. In pJOE8999, the expression of the *cas9* gene is regulated by the mannose inducible P_*manP*_ promoter from *B. subtilis*, which requires the transcriptional activator ManR and the d-mannose permease ManP (Altenbuchner 2016). Due to the absence of a ManR homologue in *E. coli*, this promoter exhibits little to no activity, which consequently minimizes the burden to the cloning host that arises from Cas9 toxicity (Sun and Altenbuchner 2010). This enabled the authors to use a high-copy pUC minimal origin of replication for *E. coli* thereby maximizing DNA yield in plasmid preparation for transformation into the target host cell (Norrander et al. 1983; Altenbuchner 2016). In contrast, alternative CRISPR-Cas systems rely on plasmids with a lower copy number in *E. coli* for successful cloning and stable maintenance (Zhang et al. 2016; Zhou et al. 2019). Although this reduces toxicity to the cloning host, it complicates transformation into the *Bacillus* target cell. Despite its benefits, the P_*manP*_ promoter also represents a drawback of the pJOE8999 system. The dependency on ManR limits its functionality in prokaryotes other than *B. subtilis* (Sun and Altenbuchner 2010). To overcome the limited host-range, two alternative CRISPR systems utilizing the well-established promoters P_*xylA*_ and P_*tetLM*_ for inducible control of Cas9 expression were developed (Toymentseva and Altenbuchner 2019). Both promoters proved to be successful for genome editing in *B. subtilis* but still depend on the presence of an inducer. This potentially interferes with expression modules in already engineered hosts and requires fine tuning of the workflow.

In this work, we expanded the pJOE8999 CRISPR-Cas9 toolkit for *Bacilli* by constructing a robust, inducer-independent, broad-host-range genome editing system with low toxicity to the cloning host. We screened constitutive promoters for control of Cas9 expression and demonstrated the versatility of the system by editing strains of the industrially relevant species *B. subtilis*, *B. licheniformis* and *B. pumilus*, by means of gene deletion, introduction of a single point mutation and integration of an expression cassette.

## Material and methods

### Bacterial strains and culture conditions

The bacterial strains and plasmids used in this study are summarized in Online Resource 1: Table S1 and Table S2, respectively.* Escherichia coli* DH10B and INV110 (Thermo Fisher Scientific, Darmstadt, Germany) were used for cloning and propagation of all plasmids. Transformation via electroporation was performed as described previously (Sambrook and Russell 2006).

*Bacillus subtilis* ATCC6051, *Bacillus licheniformis* BL01 and SH003 and *Bacillus pumilus* Jo2 were transformed using electroporation, as described by Brigidi et al. (1991) with the following modification: Upon transformation of DNA, cells were recovered in 1 mL LBSPG buffer and incubated for 60 min at 37 °C (Vehmaanperä 1989) following plating on selective LB-agar plates. Plasmid DNA was isolated from *E. coli* DH10B and was used for the transformation of *B. subtilis*, *B. licheniformis* SH003 and *B. pumilus.* In the case of *B. pumilus*, plasmid DNA was subsequently methylated in vitro with *B. pumilus* Jo2 whole cell extracts prior to transformation (Patent DE4005025). For the transformation of *B. licheniformis* BL01, plasmid DNA was isolated from *dam*/*dcm* deficient *E. coli* INV110 cells.

*B. licheniformis* SH003 was constructed from type strain DSM13 by deleting two restrictase genes using plasmids pDhsdR1 and pDhsdR2 as described elsewhere (Dutschei et al. 2022). *B. licheniformis* BL01 is deficient in its restrictase gene and the poly-gamma glutamate synthesis operon *pgsBCAE. B. licheniformis* BL01 and *B. pumilus* Jo2 were provided by BASF SE (Ludwigshafen am Rhein, Germany)*.*

*E. coli* and *Bacillus* strains were cultivated in lysogeny broth (5 g/L NaCl; Carl Roth, Karlsruhe, Germany) and appropriate antibiotics were added if necessary. Ampicillin was used at 100 µg/mL for *E. coli* and erythromycin was used at 5 µg/mL for *B. licheniformis* DSM13 derived strains. 20 µg/mL kanamycin was used for *E. coli* and *Bacillus* strains.

### DNA cloning

Oligonucleotides used in this study are summarized in Online Resource 1: Table S3 and were obtained from Thermo Fisher Scientific (Darmstadt, Germany). PCR amplification of DNA fragments for subsequent cloning was carried out with Q5 high-fidelity DNA polymerase (New England Biolabs, Frankfurt am Main, Germany) or PrimeStar GXL DNA Polymerase (Takara Bio, Saint-Germain-en-Laye, France) according to the manufacturer instructions. Taq DNA polymerase (Roboklon, Berlin, Germany) was used for colony-based PCR screening. Seamless and sequence independent cloning was performed with NEBuilder HiFi DNA Assembly Cloning Kit according to the manufacturer protocol (New England Biolabs, Frankfurt am Main, Germany). Restriction enzymes and T4 ligase were used for regular restriction (cloning) and Golden Gate (GG) cloning according to the supplier instructions (New England Biolabs, Frankfurt am Main, Germany). GG cloning was performed as described before (Radeck et al. 2017). Gene synthesis fragments (summarized in Online Resource 1: Table S4) were ordered from Geneart (Regensburg, Germany). Plasmid isolation from *E. coli* was performed using the High Pure Plasmid Isolation Kit (Roche, Mannheim, Germany). *Bacillus* genomic DNA was isolated with the High Pure PCR Template Preparation Kit (Roche, Mannheim, Germany). Crude genomic DNA from *Bacillus* for colony-based PCR screening after genome editing was isolated as described (Vingataramin and Frost 2015). DNA fragments were either isolated from an agarose gel with the Qiaquick Gel Extraction Kit (Qiagen, Hilden, Germany) or were purified using the High Pure PCR Product Purification Kit (Roche, Mannheim, Germany). DNA sequencing was conducted by Eurofins Genomics (Ebersberg, Germany) and data analysis was performed with Geneious prime (Biomatters, Auckland, New Zealand).

### Construction of initial P_*veg*_-*cas9* vectors

For the construction of the CRISPR-Cas9 vectors with sequence derivatives of the constitutively active *B. subtilis* promoter P_*veg*_, the backbone of plasmid pJOE_amyL was used. This plasmid is based on pJOE8999 (Altenbuchner 2016) and harbors the ~ 500 bp 3′ and 5′ homology regions and a spacer sequence of the *B. licheniformis* BL01 amylase gene *amyL* (complementary oligonucleotides amyL_SP_1 and amyL_SP_2) cloned into pJOE8999 via *Sfi*I and *Bsa*I, respectively. The 20 bp spacer sequence targeting the *amyL* gene was selected using the Geneious prime tool ‘Find CRISPR Sites’, with activity scoring assessed according to Doench et al. (2016) and off-target activity was analyzed against the *E. coli* DH10B genome (NCBI GenBank: NC_010473.1). In order to obtain an improved vector backbone for CRISPR-Cas9-based genome editing by preventing a possible read-through from the kanamycin selection marker, the strong terminator fragment T1T2T0 from pMUTIN2, comprising the *E. coli rrnB* T1T2 and *λ* T0 sequence (Vagner et al. 1998; GenBank accession AF072806), was integrated into pJOE_amyL upstream of the mannose inducible promoter P_*manP*_. To this purpose, the terminator fragment was amplified by PCR with oligonucleotides CC_t1t2t0_3 and CC_t1t2t0_4 using pMUTIN2 as the template. The corresponding backbone of pJOE_amyL was amplified with oligonucleotides CC_t1t2t0_1 and CC_t1t2t0_2. Both PCR products were purified and ligated using NEBuilder HiFi DNA Assembly. Sequencing of the resulting plasmid pJOE_amyL_T revealed a deviation of 4 nucleotides 34 bp upstream of the T1 hairpin from the published sequence of pMUTIN2. The obtained plasmid was used for further cloning, since no direct influence on the T1 hairpin was predicted using the RNAfold web server (http://rna.tbi.univie.ac.at/cgi-bin/RNAWebSuite/RNAfold.cgi; Gruber et al. 2008).

In a second step, the P_*manP*_ promoter for regulation of Cas9 expression was replaced with two variants of the promoter P_*veg*_, which showed significantly reduced activities compared to the parental sequence due to alterations within the −10 region (Guiziou et al. 2016). The Pv4 and Pv8 sequences, showing 20-fold and 200-fold lower expression rates compared to the parental promoter, were chosen for this purpose. Integration of fragments Pv4 and Pv8, which additionally contain the P_*fbaA*_ TSS from *B. subtilis* (GGAGAAA) and a RBS (RBS0: GATTAACTAATAAGGAGGACAAAC; Guiziou et al. 2016), was done stepwise. In an initial PCR, the oligonucleotides CC_t1t2t0_3 and CC_Pv were used to add the conserved promoter region, with the previously constructed pJOE_amyL_T serving as the template. The resulting product served as a template for a second PCR with the oligonucleotides CC_t1t2t0_3 and CC_Pv4 for Pv4 and CC_t1t2t0_3 and CC_Pv8 for Pv8, thereby introducing the core promoter sequences specific to Pv4 and Pv8. The vector backbone of pJOE_amyL_T was PCR amplified using oligonucleotides CC_t1t2t0_1 and CC_R0_cas9. The linearized backbone was then annealed with the promoter fragments of Pv4 or Pv8 using NEBuilder HiFi DNA Assembly and the resulting recombinant vectors pPv4_amyL and pPv8_amyL were transformed into *E. coli* INV110. Sequencing of the recovered recombinant plasmids revealed point mutations within almost all Pv4 and Pv8 promoter sequences. The functionality for CRISPR-Cas9-based genome editing of the 17 different pPv4_amyL and pPv8_amyL variants (named pPv4-1_amyL to pPv4-9_amyL and pPv8-1_amyL to pPv8-8_amyL; Online Resource 1: Table S5) was then analyzed by deletion of *amyL* in *B. licheniformis* BL01.

### Construction of target-specific vectors for genome editing in *Bacilli*

The applicability of the three most promising promoter sequences Pv4-5, Pv4-7 and Pv8-7 for genome editing was investigated in *B. licheniformis*, *B. subtilis* and *B. pumilus*. To enable fast and easy plasmid construction for various genomic targets, the plasmids pPv4-5_amyL, pPv4-7_amyL and pPv8-7_amyL were adapted for GG-based one-step cloning of the sgRNA spacer and homology directed repair (HDR) template. A first cloning step was carried out to combine the *Bsa*I-*lacZ* cloning site of pJOE8999 for the integration of the sgRNA spacer sequence with a GG-compatible cloning module for *Bsa*I-based cloning of the HDR template. The cloning module is based on pBSd141R (accession number: KY995200; Radeck et al. 2017) and consists of a constitutively expressed *mRFP* flanked by *Bsa*I restriction sites. *mRFP* is replaced by the HDR template in GG-based cloning and enables combined screening together with *lacZ* for spacer insertion, which greatly simplifies identification of positive clones. The GG-compatible cloning module was ordered as a synthetic fragment with flanking *Sfi*I restriction sites (Online Resource 1: Table S5; Syn_1) and was subsequently cloned into *Sfi*I digested pJOE8999, resulting in plasmid pJOE_GG. A fragment containing the sgRNA spacer *Bsa*I-*lacZ* cloning site and the GG-compatible cloning module was then PCR-amplified with oligonucleotides CC_GG_1 and CC_GG_2 and cloned via NEBuilder HiFi DNA Assembly into pPv4-5_amyL, pPv4-7_amyL and pPv8-7_amyL, which were linearized by PCR using oligonucleotides CC_GG_3 and CC_GG_4. The resulting plasmids pPv4-5_GG, pPv4-7_GG and pPv8-7_GG were used for the GG-based construction of target-specific CRISPR-Cas9 plasmids.

### Construction of* B. licheniformis hag* deletion plasmids pPv4-5_hag, pPv4-7_hag, pPv8-7_hag

The 20 bp spacer sequence targeting the *hag* gene was selected using the Geneious prime tool ‘Find CRISPR Sites’, with activity scoring assessed according to Doench et al. (2016) and off-target activity was analyzed against the *E. coli* DH10B genome (NCBI GenBank: NC_010473.1). The double stranded spacer sequence was assembled by annealing the complementary oligonucleotides hag_SP_1 and hag_SP_2, each carrying a 4 bp extension suitable for GG cloning, as described (Cobb et al. 2015). 5 µL of each oligonucleotide (100 µM) was added to 90 µL 30 mM HEPES buffer (pH 7.8). The reaction mixture was heated to 95 °C for 5 min, followed by annealing by cooling from 95 to 4 °C at a rate of 0.1 °C/s. The HDR template comprising 5′ and 3′ flanking regions (~ 500 bp each) of *hag* was generated by PCR with oligonucleotides hag_A_fw and hag_A_rv as well as hag_B_fw and hag_B_rv, using genomic DNA of *B. licheniformis* BL01 as a template, following fusion by overlap extension PCR with flanking oligonucleotides hag_A_fw and hag_B_rv. GG cloning of the spacer and HDR template into pPv4-5_GG, pPv4-7_GG and pPv8-7_GG was performed with restriction endonuclease *Bsa*I as described (Radeck et al. 2017). The resulting *hag* deletion plasmids were named pPv4-5_hag, pPv4-7_hag and pPv8-7_hag.

### Construction of* B. licheniformis degU32* point mutation plasmids pPv4-5_degU32, pPv4-7_ degU32, pPv8-7_ degU32

The 20 bp spacer sequence for *degU* was designed and assembled by annealing the complementary oligonucleotides degU32_SP_1 and degU32_SP_2, as described earlier. The *degU32* HDR template, including the mutations for the *degU* H12L mutation, which also introduces a *Pst*I recognition site for screening, and a silent point mutation to remove the PAM site in *B. licheniformis* BL01, was ordered as a synthetic fragment with flanking *Bsa*I sites (Online Resource 1: Table S5; Syn_2). GG cloning of the spacer and HDR template into pPv4-5_GG, pPv4-7_GG and pPv8-7_GG was performed with restriction endonuclease *Bsa*I. The resulting *degU32* point mutation plasmids were named pPv4-5_degU32, pPv4-7_degU32 and pPv8-7_degU32.

### Construction of *B. subtilis amyE* deletion plasmids pPv4-5_amyE, pPv4-7_ amyE, pPv8-7_ amyE

A fragment comprising the *amyE* spacer-sgRNA and HDR template of the 5′ and 3′ regions of the *amyE* gene of *B. subtilis* was ordered as a synthetic gene fragment with flanking *Bsa*I restriction sites (Online Resource 1: Table S5; Syn_3). GG cloning of the fragment into pPv4-5_GG, pPv4-7_GG and pPv8-7_GG was performed with restriction endonuclease *Bsa*I. The resulting *amyE* deletion plasmids were named pPv4-5_amyE, pPv4-7_amyE and pPv8-7_amyE.

### Construction of *B. subtilis aprE* deletion plasmids pPv4-5_aprE, pPv4-7_ aprE, pPv8-7_ aprE

A fragment comprising the *aprE* spacer-sgRNA and HDR template of the 5′ and 3′ regions of the *aprE* gene of *B. subtilis* was ordered as a synthetic gene fragment with flanking *Bsa*I restriction sites (Online Resource 1: Table S5; Syn_4). GG cloning of the fragment into pPv4-5_GG, pPv4-7_GG and pPv8-7_GG was performed with restriction endonuclease *Bsa*I. The resulting *aprE* deletion plasmids were named pPv4-5_aprE, pPv4-7_aprE and pPv8-7_aprE.

### Construction of sporulation factor deletion plasmids pPv4-5_spoIIE, pPv4-5_sigF, pPv4-5_sigE

Three individual fragments comprising the target-specific spacer-sgRNA and HDR templates of the 5′ and 3′ regions of the *spoIIE*, *sigF* and *sigE* genes of *B. pumilus* Jo2 were ordered as synthetic fragments with flanking *Bsa*I restriction sites (Online Resource 1: Table S5; Syn_5 (*spoIIE*), Syn_6 (*sigF*), Syn_7 (*sigE*)). GG cloning of each individual fragment into pPv4-5_GG was performed with restriction endonuclease *Bsa*I. The resulting deletion plasmids were named pPv4-5_spoIIE, pPv4-5_sigF and pPv4-5_sigE.

### Construction of genomic integration plasmid pPv4-5_gfp

Construction of the CRISPR-Cas9-plasmid with promoter variant Pv4-5 and a P_*aprE*_-*gfpmut2* promoter reporter gene cassette for genomic integration into the *amyL* locus of *B. licheniformis* was performed in two steps. First, the promoter reporter gene cassette was assembled. The promoter sequence of the *aprE* gene (P_*aprE*_) from *B. licheniformis* was PCR amplified with oligonucleotides PaprE_fw and PaprE_rv, using plasmid pCB56C (Wilson et al. 1994) as the template. Assembly of the P_*aprE*_ fragment with a GFPmut2 gene variant (GenBank accession number AF302837; Bartilson et al. 2001), which was ordered as a gene synthesis fragment with flanking *Bpi*I restriction sites (Online Resource 1: Table S5; Syn_8), into plasmid p689_GG was performed by GG cloning using restriction endonuclease *Bpi*I. The plasmid p689_GG, derived from pUC57, is designed for GG-based cargo integration. It comprises a *lacZ*-alpha fragment flanked by *Bpi*I restriction sites, which is in addition flanked upstream by the λ T0 terminator and downstream by the terminator sequence T_*aprE*_ of the *aprE* gene of *B. licheniformis* DSM13 and T1 of the *E. coli rrnB* gene, and was ordered as a synthetic construct (Online Resource 1: Table S5; Syn_9). The terminators allow for the transcriptional isolation of the reporter cassette after genomic integration. The resulting plasmid was named p689_gfp.

The reporter cassette in p689_gfp is flanked by *Bsa*I restriction sites, which allows for subsequent and directional assembly of the reporter gene cassette with 3′ and 5′ homology regions to create the HDR template, as described for *Bacillus* SEVA siblings (Radeck et al. 2017). The GG-compatible homology regions for *amyL* were amplified by PCR using oligonucleotides amyL_A_fw and amyL_A_rv and amyL_B_fw and amyL_B_rv, respectively. Cloning of the HDR template components and the 20 bp spacer sequence for *amyL* (complementary oligonucleotides amyL_SP_1 and amyL_SP_2) into pPv4-5_GG was performed with the restriction endonuclease *Bsa*I in a one-step reaction. The resulting plasmid was named pPv4-5_gfp.

### Genome editing in *Bacilli*

Up to 3 µg of the corresponding CRISPR-Cas9-plamids was used to transform *B. licheniformis*, *B. subtilis* and *B. pumilus* cells, following plating onto selective LB-agar plates and overnight incubation at 33–37 °C. While 37 °C was used as the standard temperature for incubation of transformation plates for *B. licheniformis* and *B. pumilus*, 33 °C was selected for incubation of *B. subtilis* ATCC6051 to prevent colonies from overgrowing due to the large colony size of this strain. The next day, 20 colonies of each transformation were subjected to colony PCR to analyze for successful CRISPR-Cas9-based modification. To this purpose, each colony was cultivated overnight in LB shaking at 37 °C and additionally transferred to fresh LB agar plates without antibiotic selection for plasmid curing at 50 °C overnight. The next day, crude genomic DNA was isolated from the overnight culture as described by Vingataramin and Frost (2015). Briefly, 125 µL overnight culture was treated with 220 µL extraction solution (240 mM NaOH, 74% EtOH, 2.7 mM EDTA, 0.45 mg/mL lysozyme, 0.45 mg/mL RNase) for 45 min at 37 °C and 30 min at 80 °C and 1000 rpm. Released crude DNA was centrifuged for 10 min at 13,000 rpm and 4 °C, and the pellet was dried at 60 °C. Finally, the crude DNA was dissolved in 10 mM TRIS–HCl pH 8.5 and served as a template for colony PCR. Colony PCR was performed for *B. licheniformis* using oligonucleotides amyL_Sc_1 and amyL_Sc_2 or analysis of *amyL* and oligonucleotides hag_Sc_1 and hag_Sc_2 for *hag*. For the deletion of *amyL*, additional screening for an alpha-amylase negative phenotype on agar plates containing 1.0% (w/v) starch, followed by staining with Lugol’s iodine, was carried out. The *degU32* point mutation was analyzed using oligonucleotides degU_Sc_1 and degU_Sc_2, following digestion of the product with *Pst*I. PCR-based screening of *B. subtilis* was done with oligonucleotides amyE_Sc_1 and amyE_Sc_2 (*amyE*) and aprE_Sc_1 and aprE_Sc_2 (*aprE*), respectively. For *B. pumilus*, oligonucleotides sigE_Sc_1 and sigE_Sc_2 were used for analysis of *sigE*, oligonucleotides sigF_Sc_1 and sigF_Sc_2 for *sigF* and oligonucleotides spoIIE_Sc_1 and spoIIE_Sc_2 for *spoIIE.* The efficiency of CRISPR-Cas9-based genome editing for each plasmid was calculated as the percentage of successful gene modifications relative to the total number of clones analyzed, based on the size of the specific PCR-amplicon or on starch hydrolysis. Except for the deletion of *amyL* in *B. licheniformis* BL01 for the analysis of all initial Pv4 and Pv8 plasmid variants and the deletions in *B. pumilus* (*n* = 1), all analyses were done in triplicates (*n* = 3). To finalize mutant construction and ensure purity of the genomic background after CRISPR-Cas9-based genome editing in *B. subtilis* and *B. licheniformis* SH003, curing of the plasmid was performed. Subsequently, a clone with the desired genomic modification was selected from the first curing agar plate and streaked onto a fresh LB agar plate to obtain single colonies, then incubated overnight at 50 °C for further plasmid curing. Single clones were tested for successful plasmid curing by replica plating onto LB and selective LB agar plates. Finally, one plasmid-cured subclone was stored as a glycerol stock and the genomic modification was validated by PCR and sequencing of the PCR product (see Online Resource 1: Fig. S1 for protocol type workflow). Statistical analyses of transformation and editing efficiencies of plasmids with promoter variants Pv4-5, Pv4-7 and Pv8-7 in *B. licheniformis* BL01 and *B. subtilis* were performed for each target gene using one-way ANOVA followed by Tukey’s post hoc test for multiple pairwise comparisons (*p* < 0.05 was considered significant) with Prism 11.02 (GraphPad, Boston, USA).

### Microscopy

Fluorescence microscopy experiments were performed using a Zeiss Imager.M2 equipped with a Plan Apochromat ×100/1.40 oil objective, a HXP-120 lamp, Axiocam 705 m camera and Zeiss ZEN software 3.12. 0.5–1 µL of bacterial culture was spotted onto a microscope slide coated with a 1.5% agarose film and sealed with a glass coverslip. Images were analyzed using the ZEN software.

### Relative gene expression analysis

#### Cultivation

*B. licheniformis* BL01 cells carrying one of the three plasmid variants pPv4-5_amyL, pPv4-7_amyL or pPv8-7_amyL with promoters Pv4-5, Pv4-7, Pv8-7 or the original pJOE8999 plasmid were cultured in shake flasks containing 30 mL of LB medium supplemented with 20 µg/mL kanamycin at 33 °C and 250 rpm. All liquid cultures were inoculated from overnight cultures to an initial OD_600nm_ of 0.05. For the original pJOE8999 plasmid, P_*manP*_-controlled *cas9* expression was induced at an OD_600nm_ of 0.5 by the addition of 0.2% d-mannose. *B. licheniformis* BL01 cells with pJOE8999, to which no d-mannose had been added (non-induced), were used as a control. All cultivations were carried out in triplicate.

Samples for qPCR analysis were collected within the logarithmic growth phase approximately 1.5 h after the induction of *cas9* expression from plasmid pJOE8999. For each sample, 1.5 mL culture volume was harvested on ice and all samples were centrifuged for 5 min at 4 °C and 13,000 rpm. The supernatant was discarded and the cell pellet was resuspended in 200 µL RNAlater solution (Thermo Fisher Scientific, Darmstadt, Germany) and stored at −80 °C.

#### RNA isolation

Total RNA of *B. licheniformis* BL01 was isolated using the peqGold Bacterial RNA Kit (Peqlab, Erlangen, Germany). To this purpose, cell pellets were resuspended in Lysis buffer T and mechanical cell disruption was performed using a bead beating grinder (MP Biomedicals, Eschwege, Germany) at 6.5 m s^−1^ for 45 s. The bead beating tubes contained glass beads (0.10–0.11 mm diameter, Sartorius, Göttingen, Germany) and acidic phenol chloroform isoamylalcohol (50:48:2). RNA was separated from cell debris by centrifugation for 5 min and 13,000 rpm at 4 °C. The RNA-containing phase was taken for subsequent isolation and purification with the peqGold Bacterial RNA Kit according to the manufacturer instructions. DNA digestion was performed with the peqGold DNaseI Digest Kit (Peqlab, Erlangen, Germany). Purity and concentration of the RNA was determined using the Nanodrop ND 1000 UV/Vis spectrophotometer (Peqlab, Erlangen, Germany). Additionally, the quality of all RNA samples for qPCR analysis was verified using the Agilent 2100 Bioanalyzer (Agilent Technologies, Waldbronn, Germany).

#### Reverse transcription quantitative PCR (RT-qPCR)

RNA standards were generated for the efficiency-corrected RT-qPCR analysis. To this purpose, PCR reactions for four gene fragments (*kanR*, *repF*, *cas9* and 16S *rRNA* gene) were performed using Primers kanR_T7_fw and kanR_rv (*kanR*), repF_T7_fw and repF_rv (*repF*), cas9_T7_fw and cas9_rv (*cas9*) with pJOE8999 as the template and rRNA_T7_fw and rRNA_rv (16S *rRNA* gene) with *B. licheniformis* BL01 genomic DNA. The PCR products were then purified from an agarose gel. The HiScribe T7 Quick High Yield RNA Synthesis Kit (New England Biolabs, Frankfurt am Main, Germany) was used for in vitro RNA transcription, following the protocol for short fragments below 0.3 kb. As recommended, all reactions were incubated for 16 h at 37 °C. After DNase treatment, the concentration and purity of all RNA standards was verified by measurements with the NanoDrop ND 1000 UV/Vis spectrophotometer.

RT-qPCR analysis was carried out with the Luna Universal One-Step RT-qPCR Kit inside the LightCycler 96 instrument (Roche, Mannheim, Germany). One RT-qPCR reaction comprised a total volume of 20 µL and contained Luna Universal One-Step Reaction Mix (2x), Luna WarmStart RT Enzyme Mix (20x), forward and reverse primers (10 µM each) and 1 µL template RNA. The RT-qPCR protocol included the reverse transcription at 55 °C for 10 min followed by an initial denaturation step at 95 °C for 1 min. Subsequently, a loop of 35 cycles including a denaturation (95 °C, 10 s) and an extension step (60 °C, 30 s) was carried out before running the melting curve in a temperature range between 60–95 °C. The primers kanR_fw and kanR_rv (*kanR*), repF_fw and repF_rv (*repF*), cas9_fw and cas9_rv (*cas9*) and rRNA_fw and rRNA_rv (16S *rRNA* gene) were used.

#### Statistical analysis

RT-qPCR runs were performed for all triplicate samples and relative gene expression levels were calculated using the efficiency-corrected relative quantification model described before (Pfaffl 2001). The efficiency-corrected relative expression ratio of each target gene was calculated for each biological replicate relative to the non-induced pJOE8999 as a control and normalized against the internal reference 16S *rRNA* gene. Normalized data was converted to relative expression as described (Pfaffl 2001) and log2 values were used for further analysis with one-way ANOVA followed by Tukey’s post hoc test for multiple pairwise comparisons (*p* < 0.05 was considered significant). Data visualization and statistical analysis were performed using Microsoft Excel and Prism 11.02 (GraphPad, Boston, USA).

## Results

### Selection of a promoter for an inducer-independent Cas9 expression system

In order to construct an inducer-independent CRISPR-Cas9 genome editing system, we used the pJOE8999 single-plasmid system as the starting point, because it demonstrated robust performance in our hands and in previous studies (Altenbuchner 2016; Schilling et al. 2018; Toymentseva and Altenbuchner 2019; Ferrando et al. 2023). To evaluate the suitability of a constitutive promoter for expression of the *cas9* gene and to develop a one-step CRISPR genome editing workflow, we replaced the mannose-inducible P_*manP*_ promoter in the original pJOE8999 vector (Altenbuchner 2016) by sequence variants of the constitutive promoter P_*veg*_ from *B. subtilis*. The *veg* gene is recognized and constitutively expressed by the vegetative Sigma70-type sigma factor SigA in *B. subtilis*. The P_*veg*_ core promoter matches the Sigma70-type promoter consensus sequence almost perfectly with the TTGACA −35 region motif and only one mismatch in the −10 region with TACAAT instead of TATAAT (Online Resource 1: Fig. S2). This may imply that the P_*veg*_ promoter exhibits high activity during the cloning process in *E. coli*. To minimize toxicity of Cas9 in the resulting constitutive expression system, the P_*veg*_ variants Pv4 and Pv8, which showed reduced activities compared to the parental sequence, were selected from a well-characterized P_*veg*_ library (Guiziou et al. 2016). According to Guiziou et al. (2016), gene expression under control of Pv4 and Pv8 showed 20-fold and 200-fold lower expression levels, respectively. This reduction in expression is attributed to the degeneration of the −10 promoter region, while the −35 region retains the consensus sequence TTGACA.

### P_*veg*_*-cas9 *variants evolved from cloning in *E. coli*

**Fig. 1:**
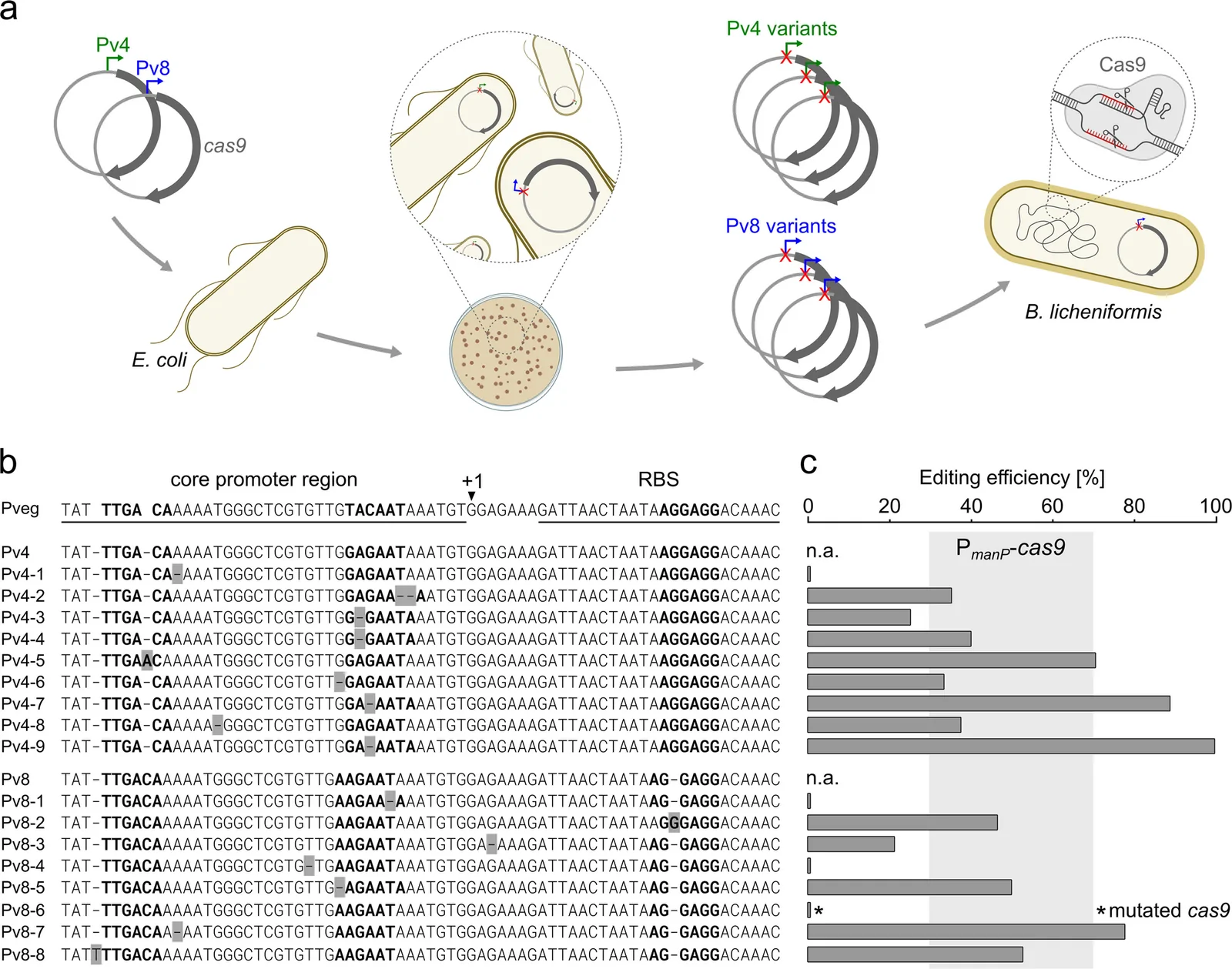
Evolved P_*veg*_-*cas9* promoter variants show different genome editing efficiencies in *B. licheniformis* BL01. **a** Cloning process in *E. coli* to replace the P_*manP*_ promoter with P_*veg*_ promoter variants Pv4 and Pv8 for Cas9 expression in pJOE8999-derivative pJOE_amyL, which targets the *amyL* locus in *B. licheniformis* BL01*.* The initially cloned Pv4 and Pv8 constructs were transformed separately into *E. coli*. The isolated plasmids with mutated Pv4 and Pv8 promoter variants were then individually analyzed and tested for their editing efficiency in *B. licheniformis* BL01. **b** Sequence alignment of the mutated P_*veg*_ promoter variants obtained from cloning of Pv4 and Pv8 promoter in front of the *cas9* gene. The complete *cas9* gene and the promoter region were analyzed by sequencing. The relevant core promoter region and the 5′ UTR region is depicted. The −35 and −10 region and the Shine Dalgarno sequence are highlighted in bold. The transcriptional start site “ + 1” according to Guiziou et al. (2016) is marked by a closed triangle. Mismatches are highlighted in grey. **c** Average editing efficiency for *amyL* deletion in *B. licheniformis* BL01 (*n* = 1; ≥ 20 clones analyzed). Pv4-3 and Pv4-4 as well as Pv4-7 and Pv4-9 are independent isolates carrying the same promoter sequence, thus representing duplicates. For reference, the range of the editing efficiency using the original pJOE8999 P_*manP*_-*cas9* construct, induced with 0.2% d-mannose, in our hands is indicated in light grey. Plasmids containing the original Pv4 and Pv8 sequences were not obtained and therefore not analyzed (n.a.) but were included as references in the sequence alignment. The asterisk indicates a frameshift mutation within the *cas9* coding region in the pPv8-6_amyL construct, leading to premature translational stop codon

Exchange of the P_*manP*_ promoter by the P_*veg*_ variants Pv4 and Pv8 was conducted in the plasmid pJOE_amyL, which is based on pJOE8999 carrying a sgRNA and homology regions to delete the alpha-amylase gene *amyL* in *B. licheniformis* BL01. However, we were unable to recover *E. coli* transformants showing the original Pv4 or Pv8 promoter sequences, despite their attenuated promoter strength compared to the native P_*veg*_ promoter (Fig. 1a). Sequence analysis revealed point mutations in the Pv4 and Pv8 promoter region across all analyzed clones (Fig. 1b). The majority of the point mutations were localized within the core promoter −10 region, followed by the spacer region between the −10 and −35 boxes. To a lesser extent, mutations were also observed within the −35 region, the transcriptional start site (TSS) and the ribosome binding site (RBS). Clones with designated plasmids pPv4-3_amyL and pPv4-4_amyL as well as pPv4-7_amyL and pPv4-9_amyL carried the same mutation likely representing siblings resulting from cell division during recovery in liquid culture rather than independent mutation events. The only clone showing the correct promoter sequence in plasmid pPv8-6_amyL is characterized by a frameshift mutation within the *cas9* coding region leading to premature translational stop. In summary, no viable clone carrying a functional *cas9* expression cassette controlled by the native Pv4 or Pv8 sequence was isolated. Instead, various mechanisms were observed that cells employ to inactivate expression constructs, highlighting the strong selective pressure for low basal levels of Cas9.

Because most of the mutated promoter sequences that evolved from cloning in *E. coli* did not indicate complete loss of promoter activity per se, all constructs were screened for their editing efficiency in *B. licheniformis* BL01, targeting the alpha-amylase gene *amyL* (Fig. 1c). Successful deletion of *amyL* was analyzed by transferring individual colonies to starch-containing agar plates and PCR amplification of the *amyL* locus with subsequent sequencing. Loss of the ability to clear starch from the agar plates indicated successful deletion of *amyL*. pPv8-6_amyL carrying the *cas9* frameshift mutation was included as a negative control. As the PCR reaction failed for five of the 17 promoter variants (Online Resource 1: Fig. S3), the editing efficiencies were determined solely on the basis of loss of starch clearing on starch-containing agar plates. The amylase-negative phenotype was consistent with the results obtained from successful PCR analyses, which confirmed Cas9-mediated targeted deletion of the *amyL* gene for most of the promoter variants. However, we observed different gene deletion efficiencies depending on the promoter used to control Cas9 expression (Fig. 1c). Phenotypic and PCR-based analysis of at least 20 individual colonies per plasmid identified well and moderately efficient Cas9-expression constructs as well as four plasmids that did not result in successful genome editing. In addition, the negative control plasmid pPv8-6_amyL, the constructs pPv4-1_amyL, pPv8-1_amyL and pPv8-4_amyL were not suitable to edit the *amyL* locus. pPv4-1_amyL and pPv8-4_amyL are characterized by a 16 bp instead of a 17 bp long spacing region between the −35 and −10 motifs, whereas pPv8-1_amyL showed an altered −10 region with a deletion of the highly conserved nucleotide T_−7_. The plasmids pPv4-5_amyL, the two identical variants pPv4-7_amyL and pPv4-9_amyL, and pPv8-7_amyL showed the highest editing efficiency (> 70%, Fig. 1c), corresponding to or even exceeding the efficiencies of P_*manP*_-*cas9*-based editing, which has generally been in the range of 30 to 70% in all of our previous experiments. pPv4-5_amyL contains an insertion of an adenine within the −35 region, extending the spacing region to 18 bp and likely altering the −35 region from the canonical TTGACA sequence to TTGAAC. pPv4-7_amyL is characterized by a deletion of G_−10_ within the −10 motif, leading to GAAATA instead of the original Pv4 GAGAAT sequence, suggesting a further weakening of the P_*veg*_ promoter, originally comprising the near-consensus TACAAT −10 hexamer. Unlike pPv4-1_amyL and pPv8-4_amyL, the construct pPv8-7_amyL, which also appears to have a shorter spacing region, showed an editing efficiency of 78% and ranked third in this mutated promoter set.

### Analysis of candidate promoter variants for broader applicability

Based on the initial evaluation of P_*veg*_ promoter variants, the most promising mutated promoter variants Pv4-5, Pv4-7 and Pv8-7 were selected for further characterization through validation of the promoter strength by RT-qPCR as well as testing for broader applicability in terms of target genes and hosts. RT-qPCR analysis of logarithmic growth samples from *B. licheniformis* BL01 harboring the corresponding *amyL* targeting plasmids revealed a 10–20-fold reduced promoter strength of Pv4-5, Pv4-7 and Pv8-7 over the induced P_*manP*_ promoter as concluded from *cas9*-mRNA levels (Online Resource 1: Fig S4). For the purpose of testing for broader applicability of Pv4-5, Pv4-7 and Pv8-7, genome editing of two gene targets was investigated in two different *Bacillus* species. Deletion of the *hag* gene encoding the major flagellar protein flagellin and introduction of the single point mutation *degU32* in the response regulator DegU was analyzed in *B. licheniformis* BL01. As the Hag protein belongs to the most abundant proteins in *Bacillus*, its deletion may spare cellular resources for target protein production (Oshiro et al. 2019). The *degU32* point mutation results in an H12L amino acid exchange in DegU and is linked to increased secretion of extracellular enzymes and further cellular adaptation mechanism throughout different growth phases in many *Bacillus* species (Kunst et al. 1975; Marlow et al. 2014). The phenotypic effects of the *hag* deletion and the *degU32* mutation were not further evaluated in the context of this study. Genome editing in *B. subtilis* was tested by targeting the alpha-amylase gene *amyE* and the alkaline protease gene *aprE*. Both enzymes are secreted, and gene deletion can be screened by loss of the ability to hydrolyze starch for *amyE* or skim milk for *aprE* in agar plate-based assays.

**Fig. 2:**
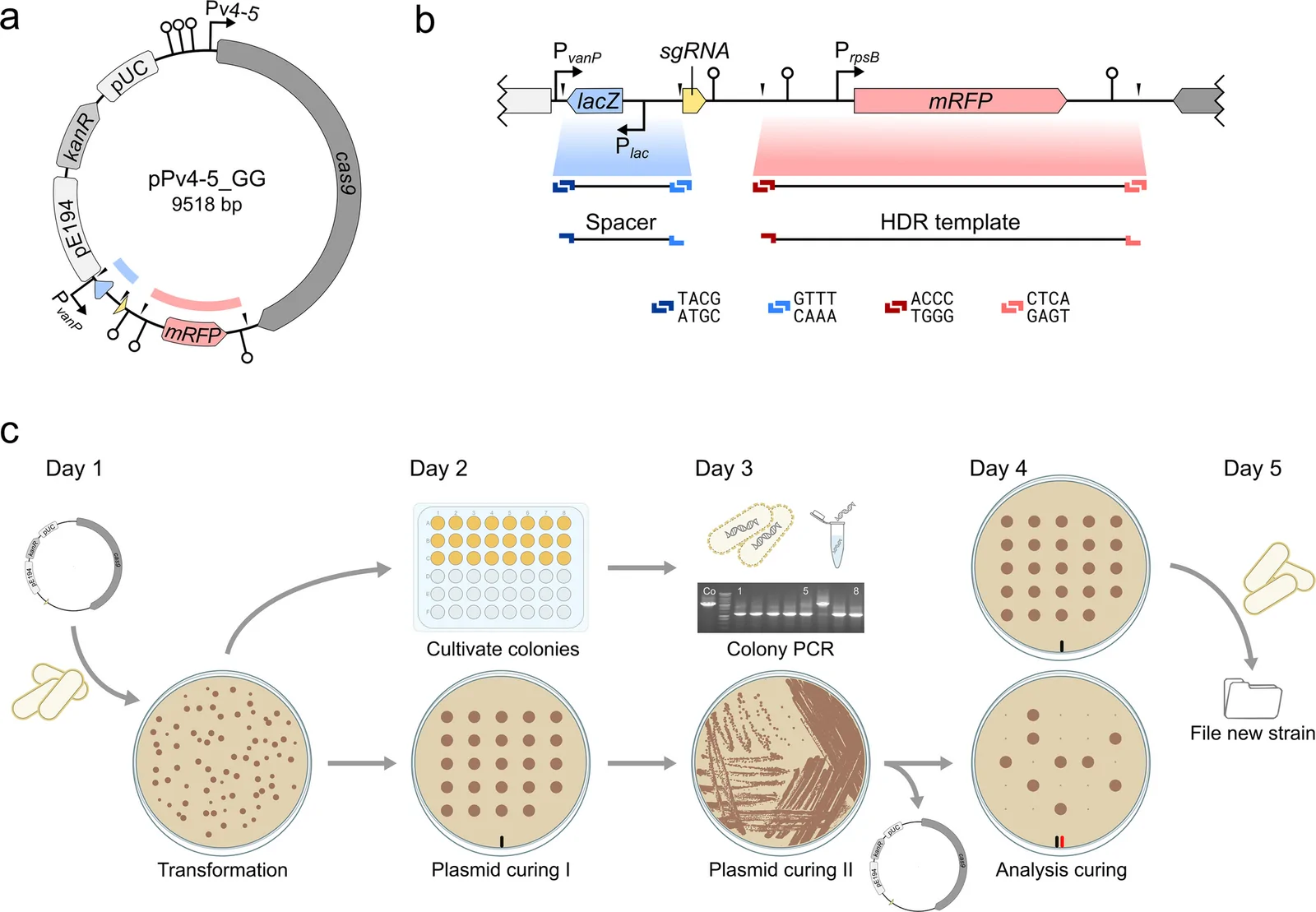
CRISPR-Cas9 vector for one-step cloning and streamlined genome editing workflow. **a** Schematic representation of the CRISPR-Cas9 vector pPv4-5_GG with the *cas9* gene under control of promoter variant Pv4-5. The plasmid backbone retains the original pJOE8999 components: pUC18 and pE194 origin of replication (light grey), Kanamycin resistance cassette (*kanR*) and *lacZ* (blue) flanked by *Bsa*I restriction sites for sgRNA spacer insertion. The sgRNA (yellow) is constitutively transcribed by P_*vanP*_. Readthrough transcription into the Pv4-5-*cas9* cassette is prevented by the upstream terminators T1T2T0. The *mRFP* (red) cloning module allows for convenient *Bsa*I-based cloning of the homology directed repair (HDR) template. *Bsa*I restriction sites are marked with triangles and Golden Gate cloning modules for *Bsa*I-based one-step-cloning of the sgRNA spacer sequence and HDR template are highlighted in blue and red, respectively. **b** Architecture of the Golden Gate cloning modules for *Bsa*I-based one-step-cloning of the sgRNA spacer sequence (blue) and HDR template (red). The 4 nt overhangs utilized for *Bsa*I-based cloning are depicted. **c** CRISPR-Cas9 workflow for rapid genome editing in *Bacillus* species. After transformation of the targeting CRISPR-Cas9 plasmid (day 1), single colonies are transferred onto agar plates for plasmid curing and into liquid culture for colony PCR (day 2). Subsequently, genomic DNA is isolated from liquid cultures and colony PCR is performed for genotype analysis. Colonies with correct genotype are then selected from first curing agar plates and streaked onto new agar plates for further plasmid curing (day 3). Single colonies are tested for loss of the plasmid by replica plating onto selective and non-selective agar plates (day 4). A pure, plasmid-free subclone carrying the desired genomic modification can be filed (day 5)

To enable fast and easy plasmid construction for the selected genomic targets, the plasmids pPv4-5_amyL, pPv4-7_amyL and pPv8-7_amyL were first adapted for Golden Gate (GG)-based one-step cloning of the sgRNA spacer and HDR template (Fig. 2a, b). The original *Bsa*I-*lacZ* cloning site of pJOE8999 for the integration of the sgRNA spacer sequence was combined with a GG-compatible *mRFP* cloning module (Radeck et al. 2017) for *Bsa*I-based cloning of the HDR template. The *mRFP* cassette is replaced by the HDR template and enables screening together with *lacZ* for spacer insertion, which greatly simplifies identification of positive clones and reduces cloning effort. The resulting plasmids pPv4-5_GG, pPv4-7_GG and pPv8-7_GG were then equipped with the spacer sequence and HDR template targeting the corresponding loci and genome editing was performed with a streamlined workflow (Fig. 2c).

**Fig. 3:**
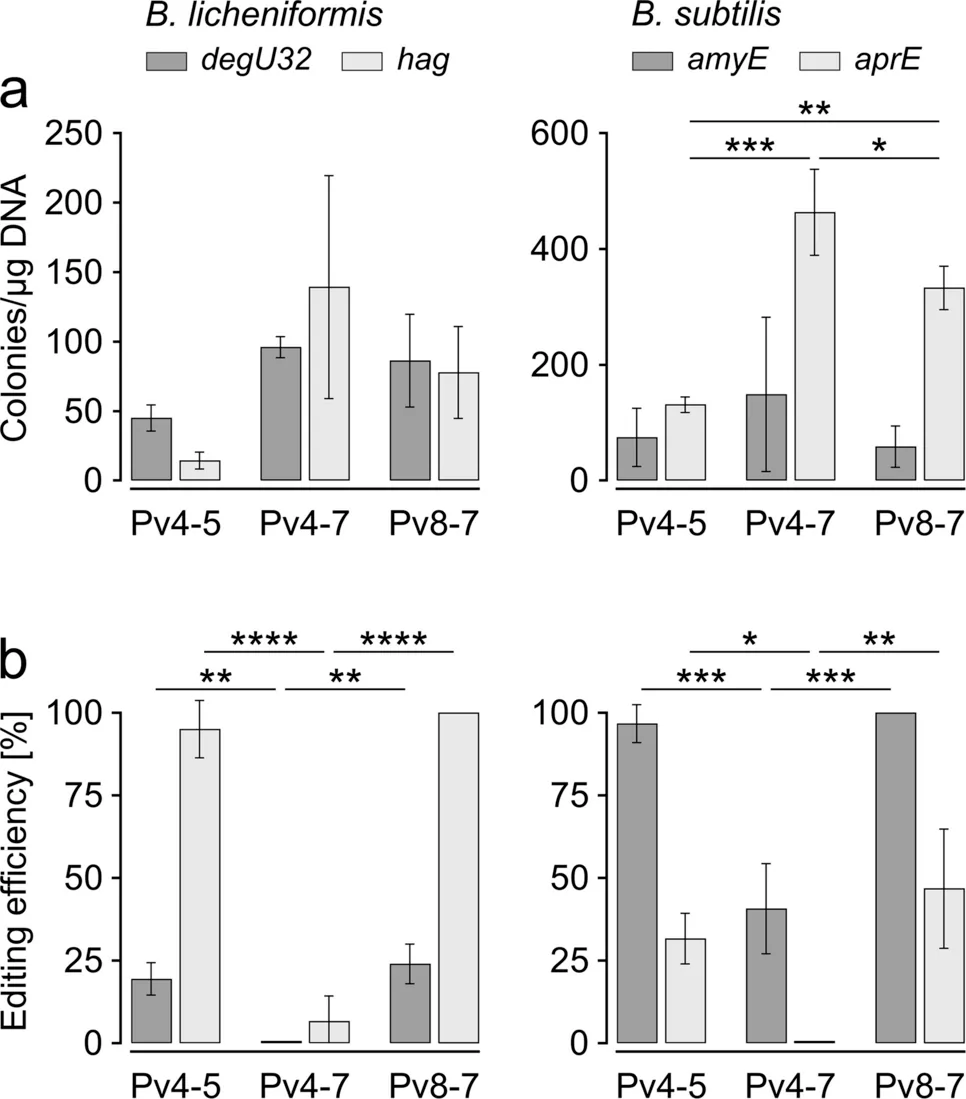
CRISPR-Cas9 system with candidate P_*veg*_ promoter variants enables broader application. The transformation efficiency (**a**) and editing efficiency (**b**) of the CRISPR-Cas9 plasmids with promoter variants Pv4-5, Pv4-7 and Pv8-7 was analyzed in *B. licheniformis* BL01 for the deletion of the *hag* gene and introducing the *degU32* single point mutation and in *B. subtilis* for deleting the *amyE* and *aprE* genes. 3 µg of plasmid DNA was transformed into *B. licheniformis* BL01 and for *B. subtilis* 1.5 µg plasmid DNA was transformed. Plated cells were incubated at 37 °C (*hag*, *amyE*) or at 33 °C (*degU32*, *aprE*) for two days. 9–20 colonies per replicate were analyzed by PCR to assess editing efficiency. Error bars indicate the standard deviation from three independent experiments (*n* = 3). Statistical differences among the plasmid variants were analyzed for each target gene by a one-way ANOVA, followed by Tukey’s post hoc test for multiple pairwise comparisons (*p*-value: * < 0,05; ** < 0,01; *** < 0,001; **** < 0,0001; only significant are shown). Note the different axis scaling in **a**

Transformation of *B. subtilis* and *B. licheniformis* BL01 was successful for all tested plasmid variants and targets (Fig. 3). Transformation plates were incubated at 37 °C or 33 °C for *B. licheniformis* and *B. subtilis,* respectively. The lower temperature for *B. subtilis* was chosen to prevent overgrowth of colonies. However, despite sufficient transformation efficiencies, modification of the *degU* gene in *B. licheniformis* BL01 at 37 °C and *amyE* in *B. subtilis* at 33 °C did not result in edited colonies for any of the promoter variants (data not shown). In contrast, adjusting the temperature to 33 °C for *degU* and 37 °C for *amyE* resulted in a 3- to 9-fold reduction in transformation efficiency, but in a significant number of correct clones for each of the plasmids as confirmed by PCR analysis (Online Resource 1: Fig. S5) and DNA sequencing to verify introduction of the *degU32* point mutation (Online Resource 1: Fig. S6a) Although the transformation efficiencies differed between all promoter variants and target genes in both strains, promoter Pv4-7 yielded the highest overall colony count, whereas Pv4-5 yielded the lowest (Fig. 3a). For plasmids targeting *degU* and *hag*, similar transformation efficiencies were observed for Pv4-7 and Pv8-7, ranging between 75 and 140 CFU per μg DNA. For the deletion of *aprE* in *B. subtilis*, the plasmids with promoter variants Pv4-7 and Pv8-7 yielded a 2- to 4-fold higher colony count compared to Pv4-5. A lower but similar transformation efficiency for all promoter variants was achieved when targeting the *amyE* gene.

Genome editing using plasmids with promoters Pv4-5, Pv4-7 and Pv8-7 for regulation of Cas9 expression resulted in a consistent ranking in both *B. subtilis* and *B. licheniformis* BL01 across all analyzed targets (Fig. 3b). Highest genome editing efficiencies were achieved using the promoter variants Pv4-5 and Pv8-7. At least 20% of all clones were positive for both promoters, independent of the strain background or the gene target. Highest editing efficiencies (> 90%) were observed for Pv4-5 and Pv8-7 for the deletions of *hag* in *B. licheniformis* BL01 and *amyE* in *B. subtilis*. In contrast, when the Pv4-7 variant was used, only 10% of the *B. licheniformis* BL01 clones and 40% of the *B. subtilis* clones showed deletion of the *hag* or *amyE* gene, respectively. Introduction of the *degU32* mutation in *B. licheniformis* BL01 and deletion of *aprE* in *B. subtilis* showed a lower success rate than editing of *hag* and *amyE* ranging from 20 to 45% positive clones. In comparison to the promoter variants Pv4-5 and Pv8-7, neither *degU* nor *aprE* was successfully edited using Pv4-7. We concluded that both genes are apparently more difficult to edit targets or the selected sgRNAs were less optimal. However, alternative protospacer sequences were not tested in the context of this work.

Throughout the primary and secondary screening of modified P_*veg*_ promoter variants, Pv4-5 and Pv8-7 showed consistently high editing efficiencies. Pv4-5 was further evaluated in *B. pumilus* by deleting the phase-II-sporulation genes *sigE*, *sigF* and *spoIIE* with editing efficiency between 45 and 55% (Online Resource 1: Fig. S7). Therefore, the promoter Pv4-5 was identified as the most reliable variant to control Cas9 expression in our adapted single-plasmid CRISPR-Cas9 system, due to its successful application in the industrially relevant *B. subtilis*, *B. licheniformis* and *B. pumilus* strains.

### Pv4-5-*cas9*-based genomic-integration in *B. licheniformis*

**Fig. 4:**
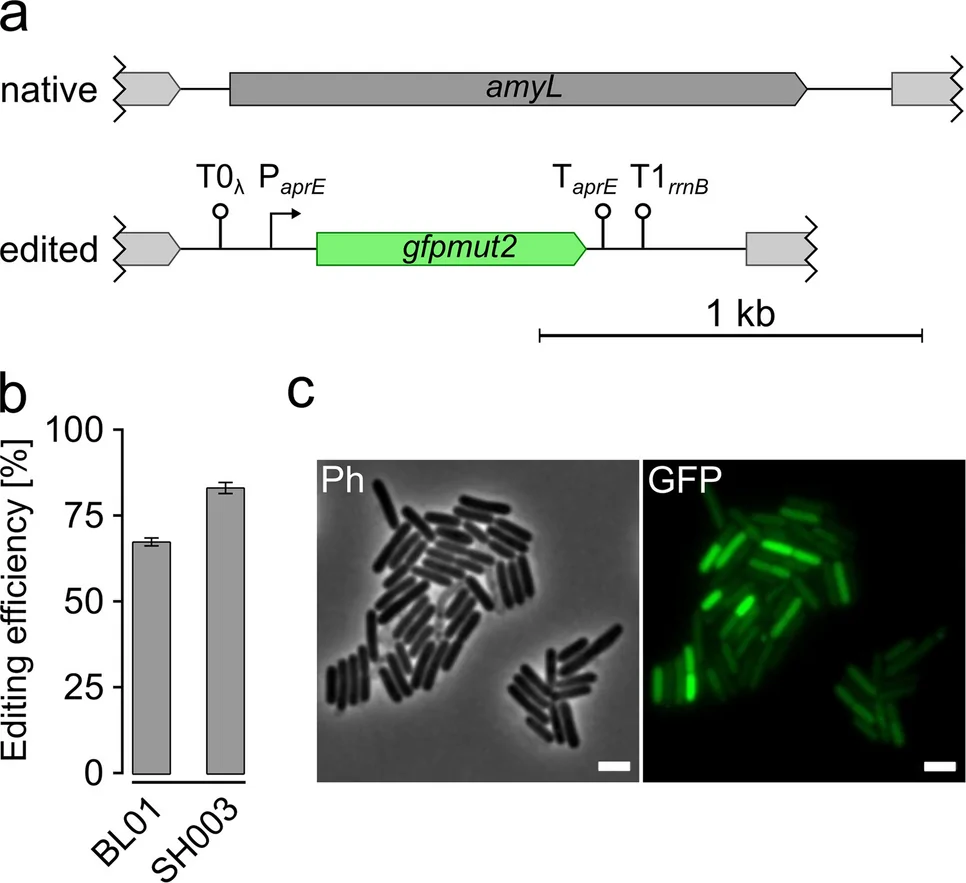
Application of the CRISPR-Cas9 system for the genomic integration of a P_*aprE*_-*gfpmut2* promoter-reporter gene fusion into the *amyL* locus of *B. licheniformis*. **a** Schema of the native and edited *amyL* locus with the integrated P*aprE*-*gfpmut2* promoter-reporter gene fusion. The P_*aprE*_ promoter sequence controlling *gfpmut2* expression and flanking terminators for the transcriptional isolation of the reporter cassette are depicted. **b** Editing efficiency of *B. licheniformis* strains BL01 and SH003 transformed with plasmid pPv4-5_gfp, comprising *cas9* under the control of promoter variant Pv4-5, an *amyL* spacer and the reporter gene cassette as a repair template. Error bars indicate the standard deviation from three independent experiments (*n* = 3). **c** Verification of the mutated strain SH003-GFP by fluorescence microscopy in LB during the late exponential phase. Representative images of phase contrast (Ph) and GFP channels are shown. Scale bar indicates 2 µm

To demonstrate the applicability of our Pv4-5-*cas9*-controlled CRISPR-Cas9 system for genomic integration, a P_*aprE*_-*gfpmut2* promoter-reporter gene fusion was integrated into the *amyL* locus of *B. licheniformis* allowing for analysis of the expression of the alkaline protease AprE (Fig. 4a). Successful integration using the plasmid pPv4-5_gfp was demonstrated for two different strains, *B. licheniformis* BL01, and *B. licheniformis* SH003, the latter derived from model strain *B. licheniformis* DSM13, with integration efficiencies of 66% and 82%, respectively, as confirmed by PCR analysis (Fig. 4b; Online Resource 1: Fig. S8). Curing of the pPv4-5_gfp plasmid was conducted for two individual clones resulting in a curing efficiency of 38% (Online Resource 1: Fig. S9b). Following sequence validation (Online Resource 1: Fig. S6b), the newly constructed strain *B. licheniformis* SH003-GFP was cultivated in LB medium and the functionality of P*aprE-gfpmut2* was confirmed by fluorescence microscopy. Samples taken during the early stationary growth phase revealed activation of the P_*aprE*_ promoter along with heterogeneity in its activity (Fig. 4c).

## Discussion

Continued advances in molecular biology tools are key to further simplifying and accelerating genetic engineering in applied industrial and academic contexts. Here, we report the optimization of the pJOE8999 single-plasmid CRISPR-Cas9 system (Altenbuchner 2016) to broaden its applicability and streamline the workflow. The original editing protocol described for pJOE8999 includes kanamycin and d-mannose in the transformation plates for plasmid selection and induction of *cas9*, thereby forcing genome editing already during outgrowth of the transformants on agar plates. This strategy represents a quasi-constitutive expression of Cas9, a common feature of many bacterial CRISPR-Cas platforms (Zhang et al. 2016; Westbrook et al. 2016; Altenbuchner 2016; Li et al. 2018; Price et al. 2019). To benefit from the usage of an *E. coli* high-copy vector while ensuring broad applicability of the promoter-*cas9* expression cassette and a streamlined workflow using a vegetative promoter for transcriptional control of the *cas9* gene, we modified the pJOE8999 system. We replaced the d-mannose inducible P_*manP*_ promoter with mutated versions of two weakened P_*veg*_ promoter variants, Pv4 and Pv8, which exhibit reduced activity compared to the native P_*veg*_ in *B. subtilis* (Guiziou et al. 2016). However, despite the anticipated 20-fold and 200-fold lower expression rates of these two modified promoter variants, we could not recover plasmids with the *cas9* gene under control of the original Pv4 or Pv8 sequence after cloning in *E. coli*. The strong selective pressure for further degeneration of the core promoter region, and the observed nonsense mutation within the *cas9* gene of pPv8-6_amyL, indicate that *cas9* is actively transcribed and represents a burden to the *E. coli* cloning host. This is consistent with a previous report on P_*veg*_ and other *Bacillus-*derived promoters showing significant transcriptional activity in *E. coli* (Li et al. 2022), as well as the frequently observed toxicity of Cas9 even at low expression levels (Toymentseva and Altenbuchner 2019; Price et al. 2019; Vento et al. 2019; Liu et al. 2020; Rostain et al. 2023). The identified mutations within the Pv4 and Pv8 promoter sequences most likely reduce the overall Cas9 expression level by decreasing predominantly transcriptional or, to a lesser extent, translational activity. Reduced transcriptional activity due to mutations in conserved core promoter elements occurred in multiple instances and affects the initial step of Cas9 expression, thus providing an effective method for minimizing the burden on the cloning host. The majority of the observed single base deletions within the spacing region between the −35 and −10 motif reduced the spacer length from 17 to 16 bp and resulted in low editing efficiency during the initial screen in *B. licheniformis* BL01. It is well known that changes in spacer length or sequence have a significant impact on recognition and anchoring by RNA polymerase (RNAP), and that promoters with a 17 bp consensus length exhibit higher transcriptional output than identical promoters with a 16 bp or 18 bp spacer (Aoyama et al. 1983; Mulligan et al. 1985; LaFleur et al. 2022; Deal et al. 2024). This general effect probably reduces the Cas9-mediated counter-selection of unedited clones during CRISPR-Cas application in *Bacilli*, resulting in lower editing efficiency. However, for promoter variant Pv8-7, Cas9 expression strength remained sufficient to allow for robust genome editing efficiencies. Mutations within the −10 box occurred more frequently in the stronger Pv4 promoter variant than in Pv8, demonstrating the potential for promoter attenuation through weakening of RNAP open complex formation, mediated primarily by the −10 motif (Feklistov and Darst 2011; Deal et al. 2024). The initial design of the P_*veg*_ library, from which the Pv4 and Pv8 variants originate, was achieved by degeneration of the −10 box and resulted in *B. subtilis* in an even distribution of expression strength compared to the sharp decline in promoter activity observed upon mutation of the −35 region (Guiziou et al. 2016). This likely indicates the strong dependence of the P_*veg*_ promoter on RNAP recognition by the canonical −35 region and a higher tolerance for changes at the −10 region, which is complemented by a current thermodynamic RNAP model for Sigma70 promoter sequences that predicts a strong interaction energy for the −35 consensus motif TTGACA, while several −10 hexamers exhibit similar interaction energies (LaFleur et al. 2022). Nevertheless, the observed deletions within the −10 sequence led to heterogeneous editing efficiencies during genome editing in the tested *Bacillus* strains, with a high degree of dependence on the exact position and the resulting change in the sequence motif. Substitutions of the highly conserved nucleotides A_−11_ and T_−7_, which are critical for base-specific interactions with the Sigma factor domain 2 during nucleation and transcription bubble formation, either resulted in low editing efficiency for the promoter variants Pv4-3 and Pv4-4, where both bases were affected, or to higher editing efficiencies, presumably due to stronger Cas9 expression, observed for Pv4-7 and Pv4-9, where A_−11_ was retained (Feklistov and Darst 2011). For the weaker Pv8 promoter variant, substitution of T_−7_ in Pv8-1 resulted in a complete loss of genome editing ability, underscoring the dependence of the entire −10 motif, required for formation and propagation of the transcription bubble (Feklistov and Darst 2011; Saecker et al. 2021; LaFleur et al. 2022). For the promoter version Pv4-5, which achieved robust genome editing efficiencies across all tested *Bacilli*, the attenuation resulted from degeneration of the consensus −35 region. The insertion of an adenine at position −31, altering the consensus motif from TTGACA to TTGAAC, weakens the base-specific interaction with residues in the helix-turn-helix motif of Sigma factor domain 4.2 (Gardella et al. 1989; Siegele et al. 1989; Feklistov and Darst 2011; LaFleur et al. 2022). This modification and the resulting extension of the spacer length from 17 to 18 bp, presumably further reducing promoter activity in *E. coli*, could be counterbalanced by a partially conserved TRTGn extended − 10 motif. As demonstrated by previous studies, the absence of a consensus −35 box or an increased spacing can be compensated for by an extended −10 box, which ensures the correct positioning of RNAP on the DNA and thus potentially maintains Cas9 expression levels for sufficient counter-selection in *Bacillus* (Campbell et al. 2002; Voskuil and Chambliss 2002; Mitchell 2003; Ruff et al. 2015). The diversity of the observed mutations underscores the necessity and possibilities of reduced Cas9 expression within the cloning host, thereby allowing tolerance to the otherwise toxic nuclease. However, upon application in *Bacillus*, only a few of these variants resulted in adequate genome editing efficiencies, emphasizing the need for finely tuned Cas9 expression levels. Cas9 expression is an important factor in the application of CRISPR-Cas in bacteria to ensure a balance between strong selection pressure through DNA cleavage and the elimination of unedited clones, on the one hand, and homologous repair, on the other hand. As observed here for promoter variant Pv4-7 in *B. subtilis* and *B. licheniformis*, as well as in several other studies, high transformation efficiencies in CRISPR-Cas applications usually result from weak nuclease-mediated counter-selection against unedited clones, leading to low overall editing efficiency (Zheng et al. 2017; Mougiakos et al. 2017; Toymentseva and Altenbuchner 2019; Price et al. 2020). Furthermore, the expression strength and targeting efficiency of the selected sgRNA, as well as the recombination efficiency, often affect the success of genome editing and likely represent bottlenecks for the difficult-to-edit targets *degU* and *aprE* in *B. licheniformis* and *B. subtilis*, respectively. Variation in temperature, as observed in our case, or the use of alternative media or sgRNAs can therefore be beneficial and lead to more efficient genome editing (Mougiakos et al. 2017; Guo et al. 2018; Price et al. 2020).

Based on the evolved Sigma70-type promoter variant Pv4-5 for Cas9 expression, we were able to develop an efficient, inducer-independent CRISPR-Cas9 single-plasmid system for fast genome editing in *Bacillus* that can be easily reconfigured through one-step GG cloning. The robustness of this adapted mutagenesis system was comprehensively validated for various markerless genomic modifications in three different industrially relevant *Bacillus* species, highlighting its broad applicability for genome editing across multiple host organisms.

## Supplementary Information

Below is the link to the electronic supplementary material.

ESM 1 (PDF 17.9 MB)

## Data Availability

All data generated or analyzed during this study are available in the article and its Supplementary Information, including Supplementary Figures and Supplementary Tables. Strains *Bacillus licheniformis* DSM13 and *Bacillus subtilis* ATCC6051 as well as plasmids used for editing these strains are available from the corresponding authors upon request. Strains *Bacillus licheniformis* BL01 and *Bacillus pumilus* Jo2 as well as plasmids specific for these strains are proprietary industrial materials and are not publicly available due to commercial restrictions. Access may be granted for non-commercial research purposes under a material transfer agreement (MTA), subject to internal approval.
