# Supplementary material for: An inducer-independent, single-plasmid CRISPR-Cas9 system for genome editing in Bacillus species
Source: Appl Microbiol Biotechnol. 2026 Sep 11;110:278. doi: 10.1007/s00253-026-14030-6 (PMC13582722; doi:10.1007/s00253-026-14030-6)
Supplement: Supplementary file 1 [file 253_2026_14030_MOESM1_ESM.pdf]

Supplementary information Online Resource 1

Applied Microbiology and Biotechnology

An inducer-independent, single-plasmid CRISPR-Cas9 system for genome editing in *Bacillus* species

Maximilian Hilkmann[1,2], Norma Welsch[1,2], Max Fabian Felle[3], Thomas Schweder[1,2*], Mathis Appelbaum[1,2,3*]

[1]Pharmaceutical Biotechnology, Institute of Pharmacy, University of Greifswald, 17487 Greifswald, Germany

[2]Institute of Marine Biotechnology, 17489 Greifswald, Germany

[3]BASF SE, 67063 Ludwigshafen am Rhein, Germany

*Correspondence should be addressed to T.S. (e-mail: schweder@uni-greifswald.de) and to M.A. (e-mail: mathis.appelbaum@basf.com)

Additional figures

- Figure S1: Graphical protocol CRISPR-Cas9 workflow
- Figure S2: Nucleotide alignment P*veg*
- Figure S3: PCR analysis *amyL* deletion in *B. licheniformis* BL01 with Pveg variants
- Figure S4: Analysis of the relative gene expression levels for Cas9 controlled by Pv4-5, Pv4-7 and Pv8-7 by RT-qPCR
- Figure S5: PCR analysis genome editing *B. licheniformis* BL01 and *B. subtilis*
- Figure S6: Confirmation of modified *amyL* and *degU* loci in *B. licheniformis* BL01 and SH003 by DNA sequencing
- Figure S7: *Bacillus pumilus* Jo2 genome editing efficiency
- Figure S8: Gel images integration of reporter-fusion in *B. licheniformis* SH003
- Figure S9: Analysis of plasmid curing efficiency

Additional tables

- Table S1: Bacterial strains
- Table S2: Plasmids
- Table S3: Oligonucleotides
- Table S4: Nucleotide sequences of gene synthesis fragments
- Table S5: Nucleotide sequences of mutated Pv4 and Pv8 promoter variants

Workflow for CRISPR-Cas9 genome editing in *Bacillus* species

Day 1
Transformation & Cas9 expression

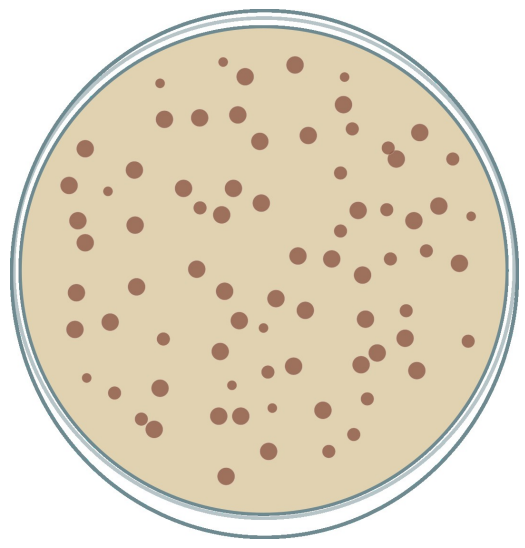

- plate transformation mix onto LB (Lennox) with 20 µg/mL kanamycin
- incubate 1-2 days at 30-37 °C (depending on strain)

Day 2
Colony analysis & plasmid curing I

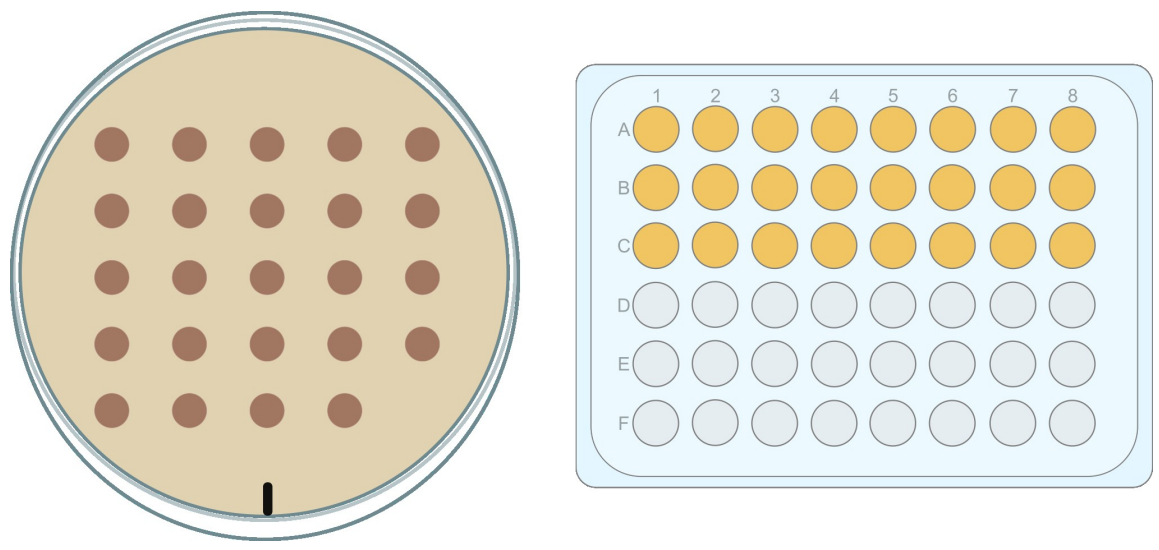

- transfer single colonies onto LB agar plate and inoculate in microwell plate (for isolation of crude genomic DNA)
- incubate agar plate overnight at 50 °C (loss of plasmid due to temperature sensitive replication)
- shake microwell plate overnight at 37 °C

Day 3
Colony-PCR & plasmid curing II

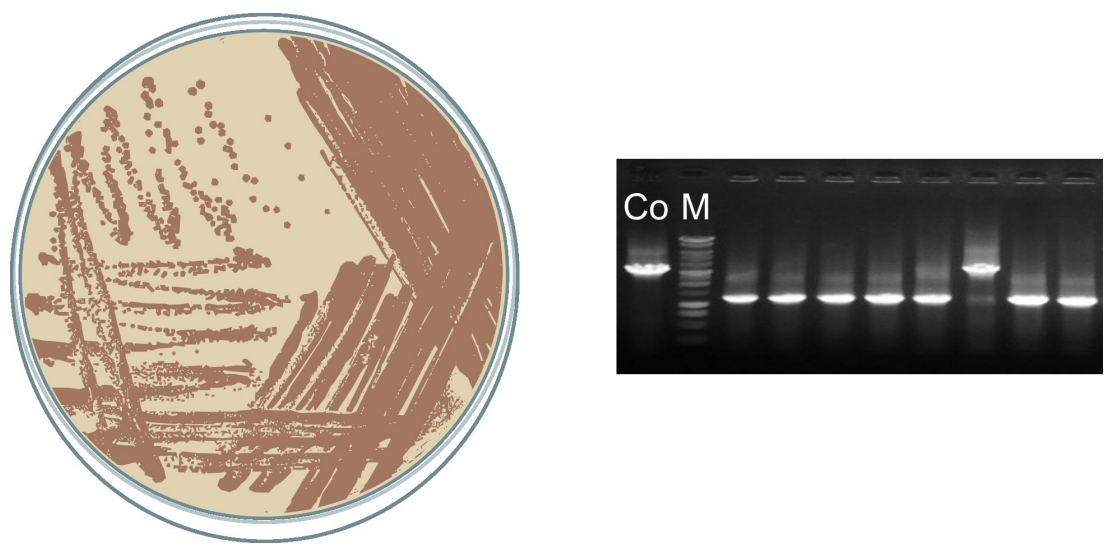

- isolate crude genomic DNA from microwell plate cultures
- PCR analysis of target gene
- streak clone with desired genomic modification onto fresh LB agar plate to obtain single clones
- incubate agar plate overnight at 50 °C

Day 4
Analysis of plasmid loss (Grid)

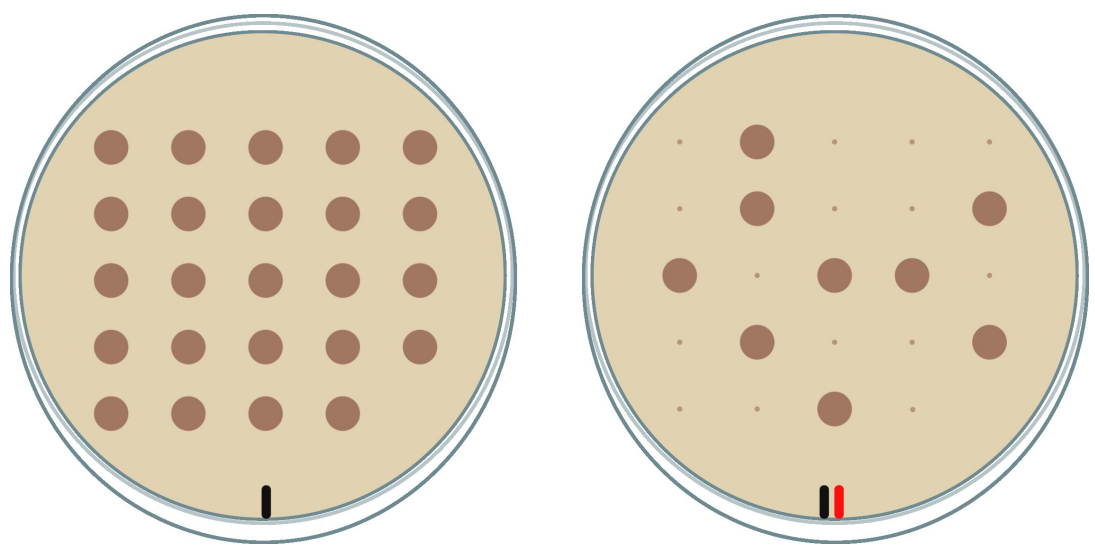

- replica plating of single clones onto LB with and without 20 µg/mL kanamycin
- incubate 1-2 days at 30-37 °C (depending on strain)

Day 5
Finalization of the new strain

- analyze Grid (kanamycin sensitive clones lost plasmid)
- inoculate kanamycin sensitive clone in LB for cryo stock
- validate genomic modification (PCR & sequencing)

Fig. S1 CRISPR-Cas9 workflow for rapid genome editing in *Bacillus* species. After transformation of the targeting CRISPR-Cas9 plasmid (Day 1), single colonies are transferred onto agar plates for plasmid curing and into liquid cultures for colony PCR (Day 2). Subsequently, genomic DNA is isolated from liquid cultures and colony PCR is performed for genotype analysis. Colonies with correct genotype are then selected from first curing agar plates and streaked onto new agar plates for further plasmid curing (Day 3). Single colonies are tested for loss of the plasmid by replica plating onto selective and non-selective agar plates (Day 4). A pure, plasmid-free subclone carrying the desired genomic modification can be filed (Day 5).

		-35	17 bp	-10	Start
E. coli Sigma70 core		TTGACA		TATAAT	
B. subtilis 168	TAATTTAAATTTTAT	TTGACA	AAAATGGGCTCGTGTTG	TACAAT	AAATGTAGTGAGGTGGATGCAATG
B. licheniformis DSM13	TAATTTAAATTTTAT	TTGACA	AAAATGGGCTTCTGGTG	TATACT	GAATATAGTGAGGTGGATGCAATG
B. pumilus ATCC7061	TAATTTAATTTTTAT	TTGACA	AAAACGGGCCGTTGTTG	TATAAT	AATCTCAGTGAGGTGGATGCAATG
B. velezensis FZB42	TAATTTAAATTTTAT	TTGACA	AAATTGGGCTCTTGTTG	TACAAT	AAATGTAGTGAGGTGGATGCAATG

Fig. S2 The core promoter region of the *veg* gene is conserved among various *Bacilli*. Nucleotide alignment of *veg* gene core promoter region from selected *Bacillus* species. *E. coli* Sigma70 consensus with the strong -35 and -10 hexamers and 17 bp spacer (LaFleur et al. 2022) is shown as a reference. The -35 and -10 hexamers are highlighted in grey and the start codon is marked with a line.

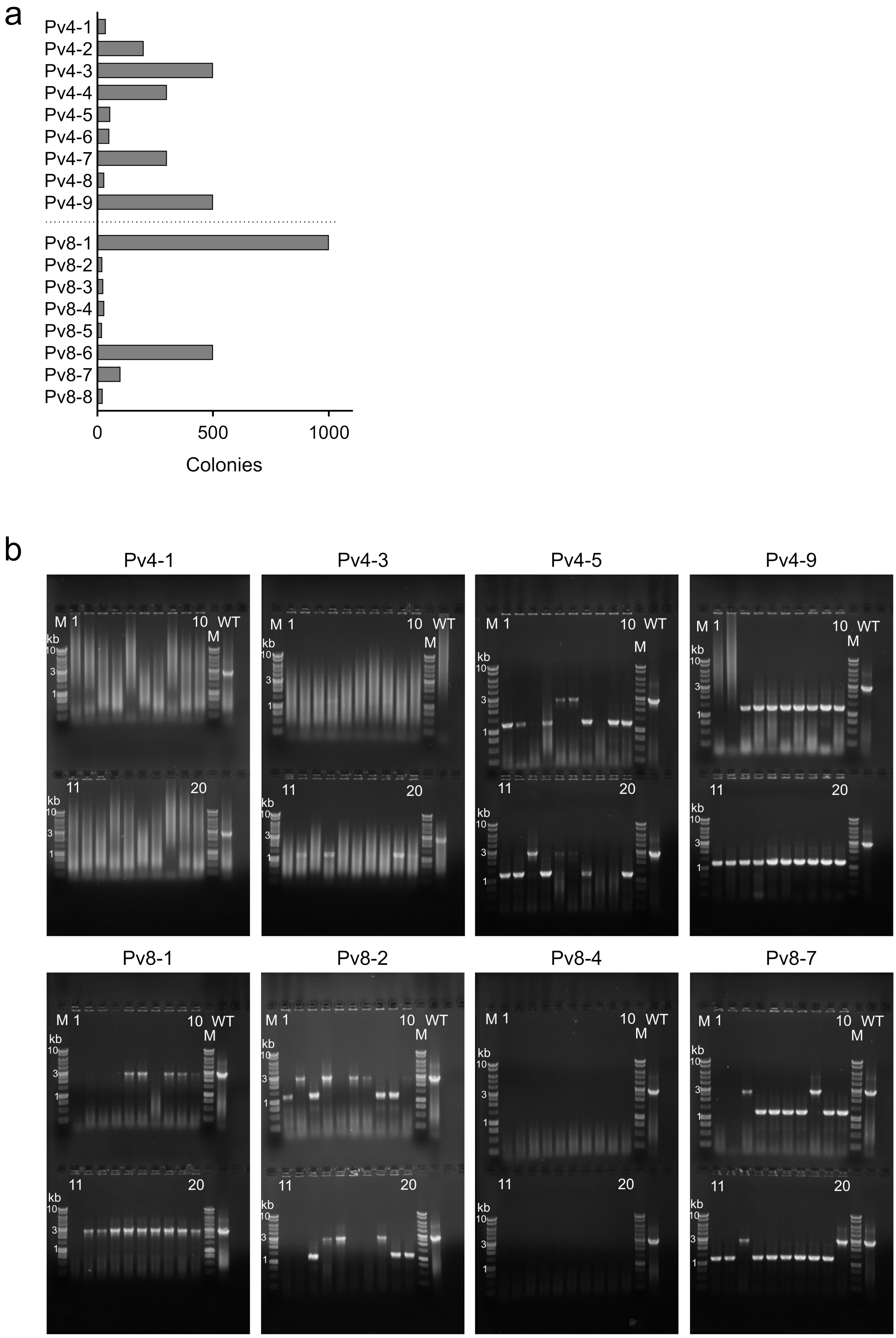

Fig. S3 Analysis of plasmid variants with evolved Pv4 and Pv8 promoter sequences for the deletion of the alpha-amylase gene *amyL* in *B. licheniformis* BL01. **(a)** Total number of colonies obtained after transformation with the plasmid variants. Where the number of colonies per agar plate exceeded 400, the count was estimated at 500 or 1,000 colonies respectively. The experiment was conducted once (n=1). **(b)** Representative gel images from the PCR-based analysis of the *amyL* deletion. PCR analysis failed for 5 of the 17 tested promoter variants (Pv4-1, Pv4-2, Pv4-3, Pv4-4, Pv8-4). The editing efficiencies shown in Fig 1c were therefore determined solely on the basis of agar plates containing starch. The amylase-negative phenotype was consistent with the results obtained from successful PCR reactions. 'Mixed' colonies showing both edited and wild type (WT) PCR fragments, were classified as WT based on observed starch hydrolysis. 20 colonies per plasmid variant were analyzed. Size of PCR fragments was ~1.2 kB for correctly edited colonies and ~2.7 kB for WT. A DNA marker (M) was used as reference.

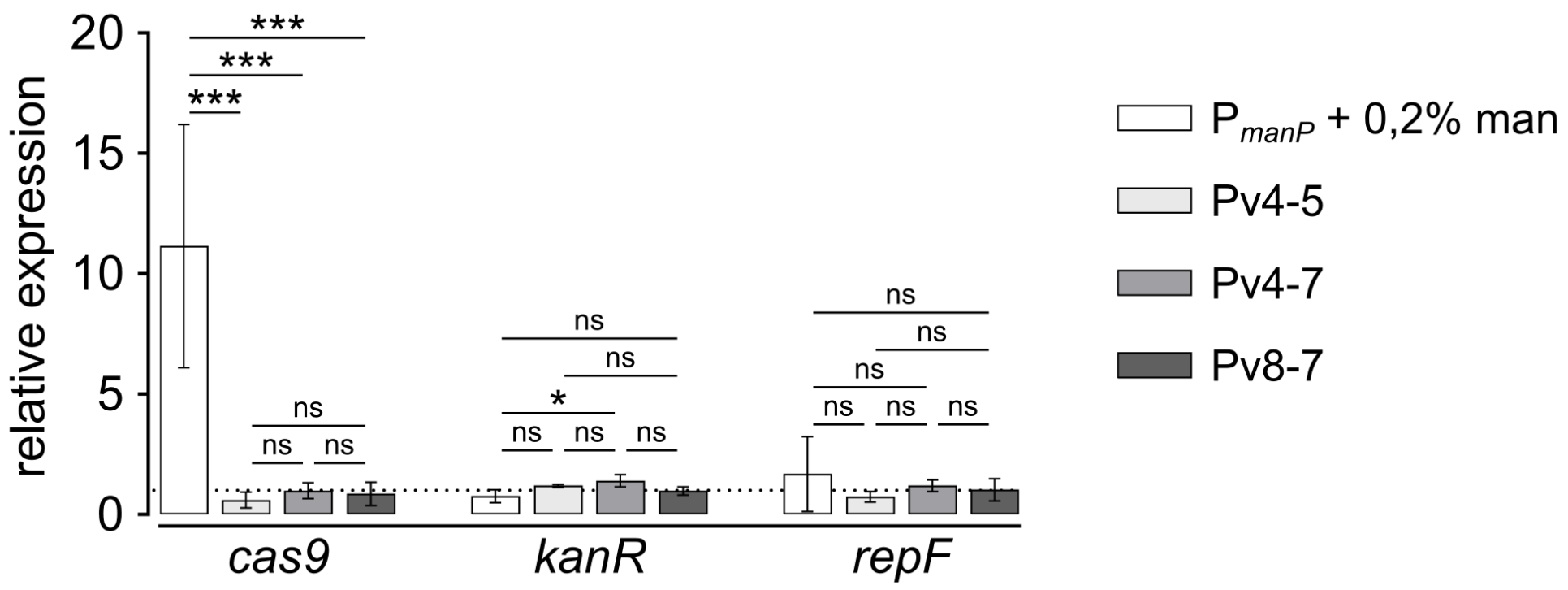

Fig. S4 Analysis of the relative gene expression levels of the plasmids with promoter variants Pv4-5, Pv4-7, Pv8-7 and the original pJOE8999 plasmid in *B. licheniformis* BL01. Strains were cultured in LB and P_{manP}-controlled *cas9* expression from pJOE8999 was induced at an OD_{600nm} of 0.5 with 0.2% D-mannose. Samples for RNA extraction and reverse transcription quantitative PCR (RT-qPCR) were taken 1.5 h after the induction in logarithmic growth phase. Cultures with pJOE8999, to which no D-mannose had been added (non-induced), served as a control for the calculation of the relative expression ratio for each target gene. Normalization was conducted against the internal reference *16S rRNA* gene. The dashed line references the gene expression levels of the non-induced control. Error bars indicate the standard deviation from three independent experiments (n=3). Statistical differences among the plasmid variants were analyzed for each gene by a one-way ANOVA, followed by Tukey's post-hoc test for multiple pairwise comparisons (p-value: * < 0,05; ** < 0,01; *** < 0,001; ns = not significant).

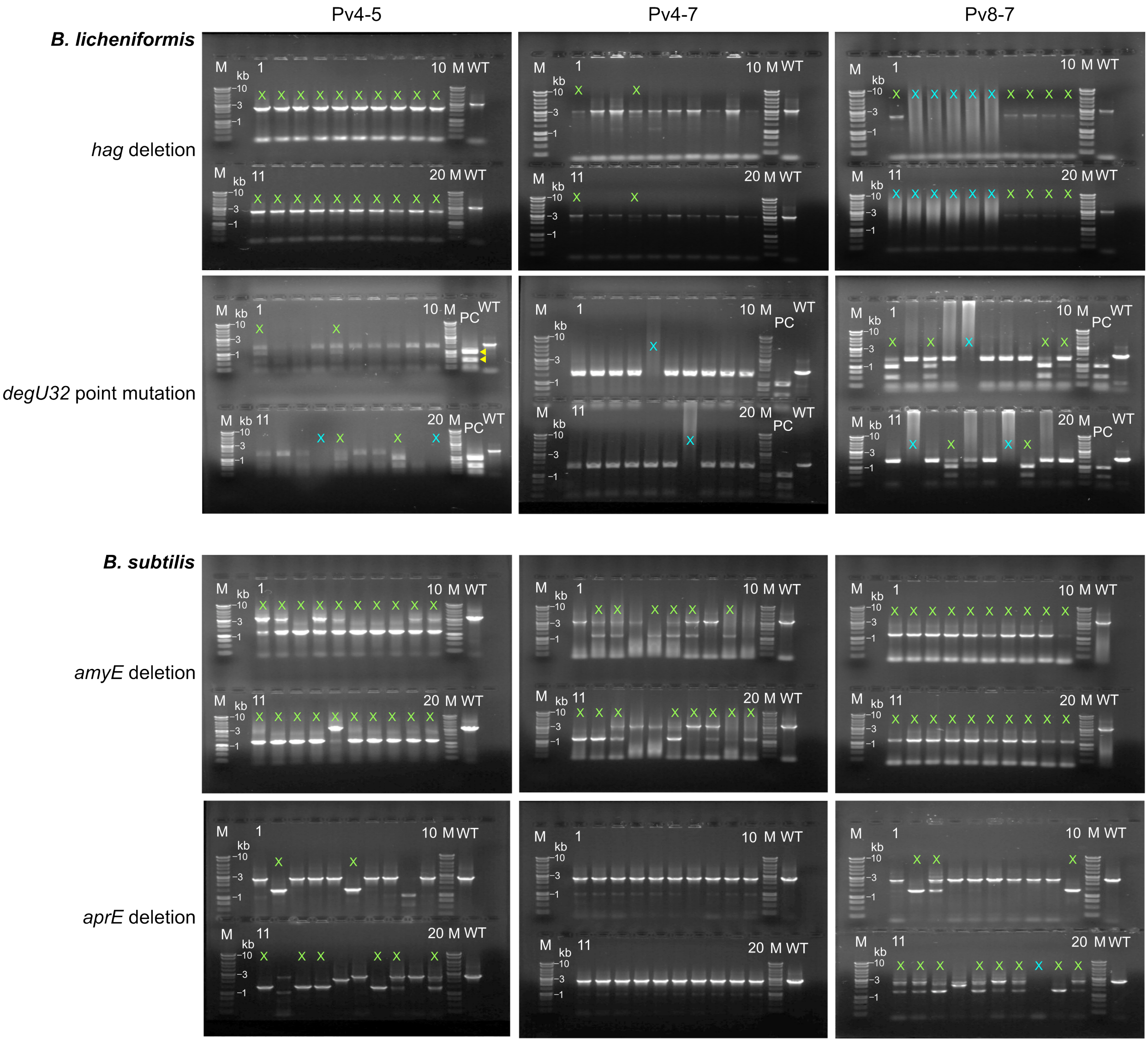

Fig. S5 PCR-based analysis of editing efficiencies of plasmids with promoter variants Pv4-5, Pv4-7, Pv8-7 for genome editing in *B. licheniformis* and *B. subtilis*. The efficiency of genome editing was assessed for the deletion of the flagellin-encoding gene *hag* and the introduction of the *degU32* point mutation in *B. licheniformis* BL01, as well as for the deletion of the *amyE* and *aprE* genes in *B. subtilis*. Representative gel images from one replicate experiment (total n=3) for each plasmid and each modification are shown. Correctly edited colonies (marked with green X) with the deletion of *hag* show a smaller PCR product (~2.3 kb) compared to the wild type (WT; ~3 kb). 'Mixed' colonies showing both edited and WT PCR fragments, were considered correctly edited for all experiments. The *degU32* point mutation results in the introduction of a *Pst*I restriction site, which enables analysis by digesting the PCR product with *Pst*I. The PCR product of the WT *degU* locus remains unaltered (~1.3 kb). The presence of both digestion fragments with a size of ~0.4 kb and 0.9 kb (marked by yellow triangle at positive control (PC)) after *Pst*I digestion was considered as correctly edited. The correct deletion of *amyE* and *aprE* in *B. subtilis* results in a PCR product of 1,4 kb (WT: 3.4 kb) or 1.5 kb (WT: 2.5 kb), respectively. Colonies with unsuccessful PCR reactions (marked with cyan X) were discarded from the analysis. 20 colonies were analyzed for each of the three replicates. A DNA marker (M) was used as reference.

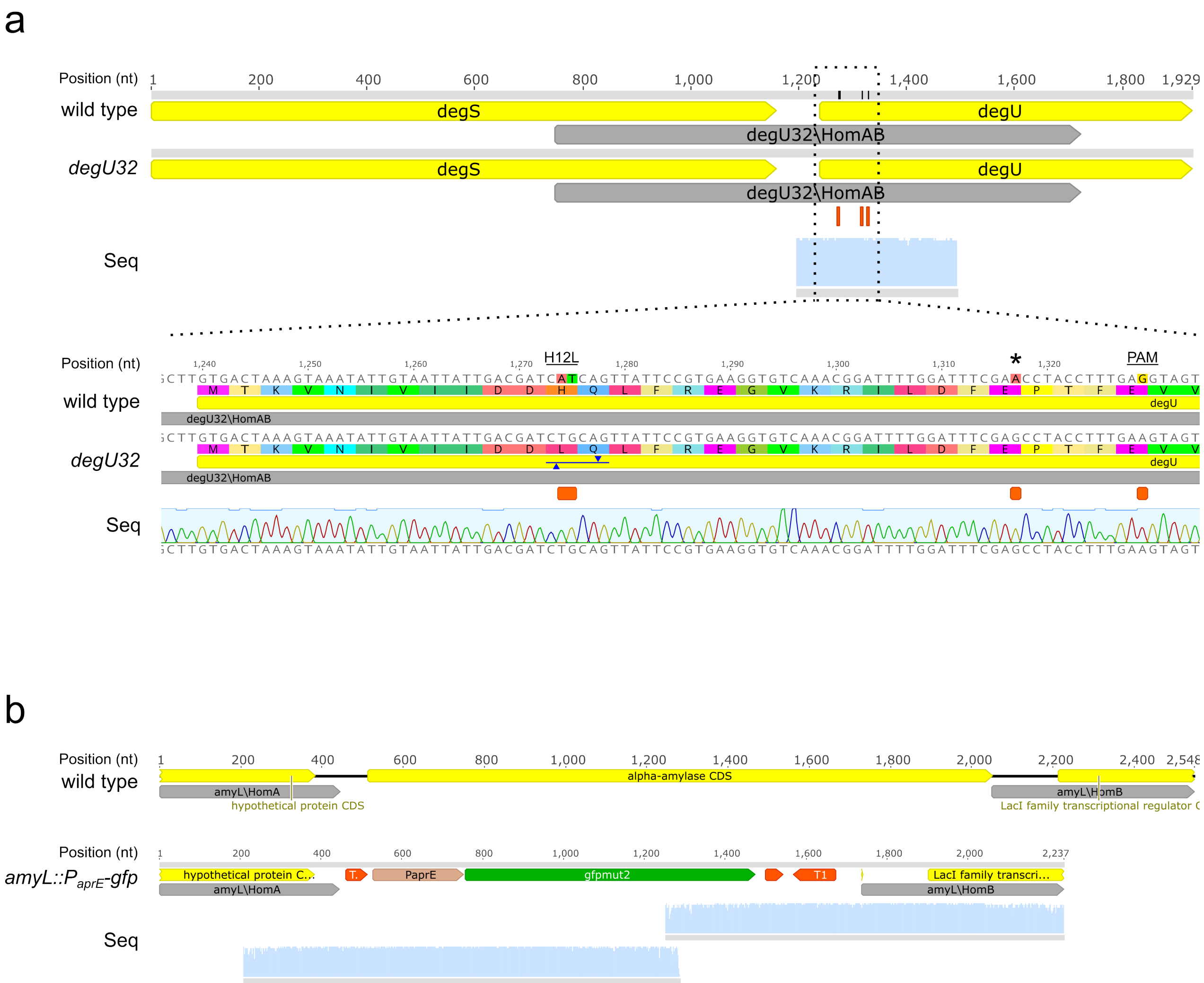

Fig. S6 Confirmation of modified loci in *B. licheniformis*. **(a)** Comparison of the wild type *degU* locus with the introduced point mutation *degU32* and a sequencing analysis of the modified locus (Seq). Base substitutions introduced by homology directed repair using the repair sequence (gray) are highlighted in red. Shown is the complete *degSU* region and a close-up of the 5'-region of *degU.* The substitution leading to the *degU32* missense mutation H12L and the introduction of a *Pst*I restriction site (marked with blue line and triangles) is indicated. The protospacer adjacent motif (PAM) 'AGG' was removed by a silent mutation and an additional silent mutation was introduced to restore codon usage (asterisk). **(b)** Comparison of the wild type and modified *amyL* locus, where *amyL* was replaced by the promoter-reporter gene fusion P_{aprE}-*gfpmut2*. Sequencing analysis of the integrated reporter-gene fusion is shown (Seq). Terminators are shown in red.

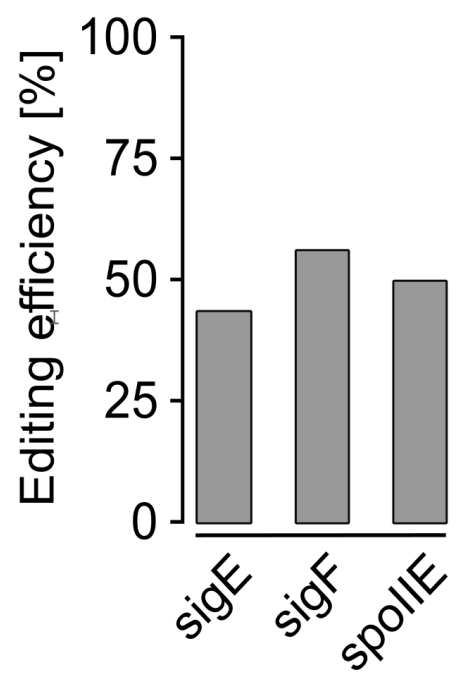

Fig. S7 The editing efficiency of the CRISPR-Cas9 plasmid with promoter variant Pv4-5 was analyzed in *B. pumilus* Jo2 for the deletion of the phase-II-sporulation genes *sigE, sigF* and *spoIIE*. 3 µg plasmid DNA was transformed. Plated cells were incubated at 37 °C for two days. 16 colonies for each deletion were analyzed by PCR to assess editing efficiency.

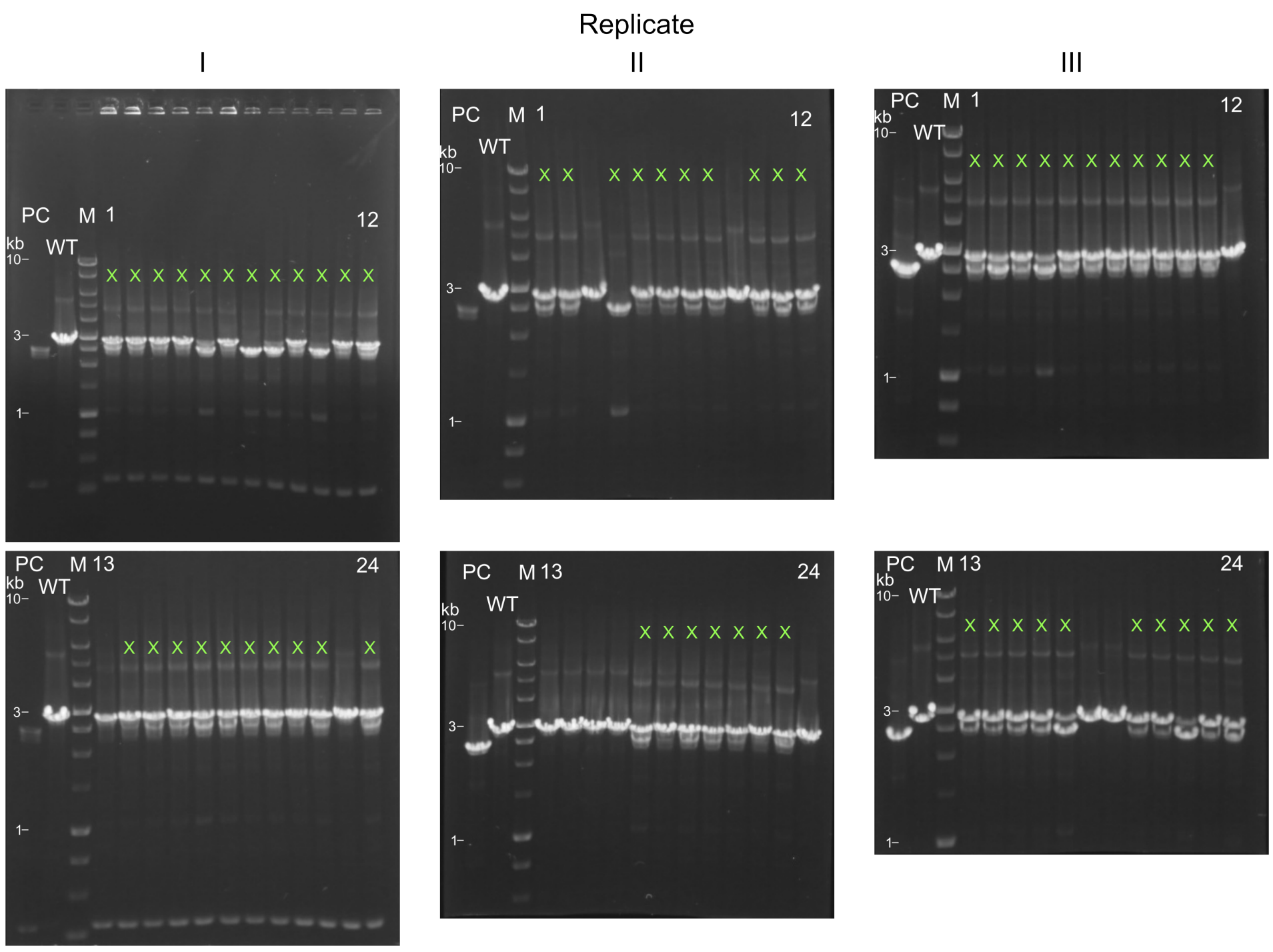

Fig. S8 PCR-based analysis of editing efficiency of the plasmid with promoter variant Pv4-5 for the integration of a promoter-reporter gene fusion into the *amyL* locus of *B. licheniformis* SH003. Gel images from each replicate experiment (total n=3) are shown. Correctly edited colonies (marked with green X) with the integration of the reporter gene fusion show a smaller PCR product (~2.5 kb) compared to the wild type (WT; ~2.7 kb). 'Mixed' colonies showing both edited and WT PCR fragments, were considered correctly edited. 24 colonies were analyzed for each of the three replicates. A DNA marker (M) and a positive control (PC) were used as references.

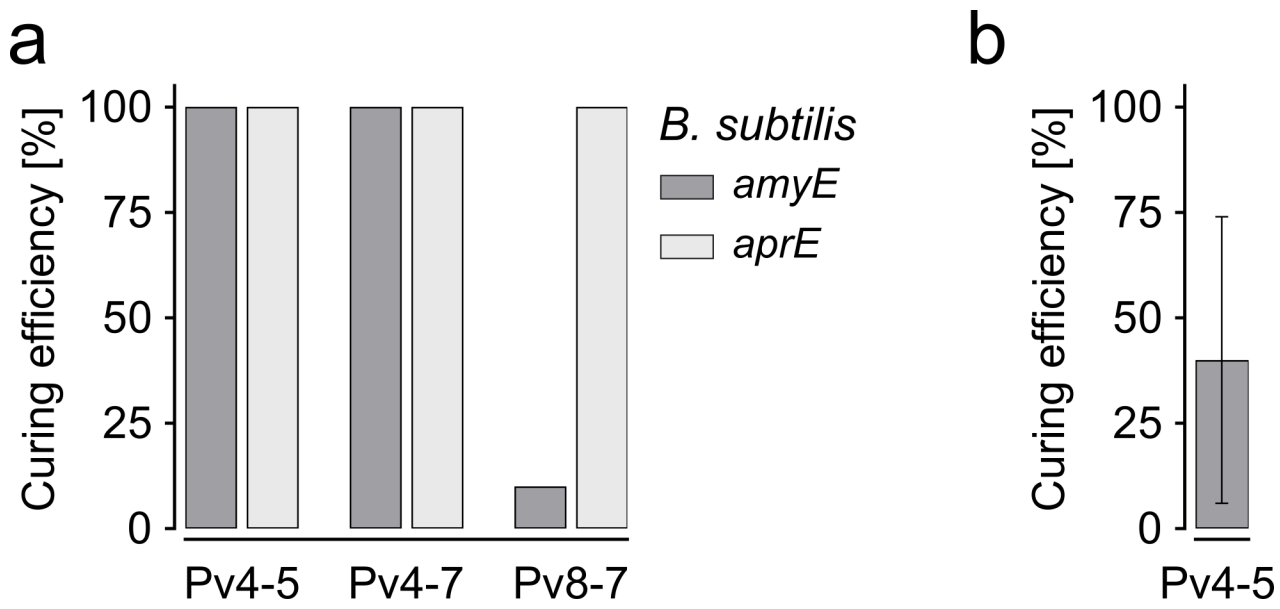

Fig. S9 Analysis of plasmid curing efficiency. **(a)** Curing efficiency of the plasmids with promoter variants Pv4-5, Pv4-7, Pv8-7 was analyzed during the deletion of *amyE* and *aprE* in *B. subtilis*. One correctly edited colony for each plasmid and each deletion (n=1) was streaked onto a fresh LB agar plate to obtain single colonies. Subsequently, 25 of these single colonies were analyzed for plasmid loss by replica plating onto LB and selective LB agar plates. **(b)** Curing efficiency of plasmid with promoter variant Pv4-5 during the integration of a promoter-reporter gene fusion into the *amyL* locus of *B. licheniformis* SH003. Two correctly edited colonies (n=2) were streaked onto LB agar plates and 25 single colonies were analyzed for plasmid loss.

Table S1: Strains used in this study

Strain	Description	Reference
E. coli DH10B	F- *endA1 recA1 galE15 galK16 nupG rpsL* Δ*lacX74* Φ80*lacZ*Δ*M15 araD139* Δ(*ara,leu*)7697 *mcrA* Δ(*mrr-hsdRMS-mcrBC*) λ-	Thermo Fisher Scientific, Darmstadt, Germany
E. coli INV110	F' {*tra*Δ*36 proAB lacI*^{q} *lac*Δ*M15*} *rpsL*(StrR) *thr leu endA thi-1 lacY galK galT ara tonA tsx dam dcm supE44* Δ*(lac-proAB)* Δ*(mcrC-mrr)102::Tn10*(TetR)	Thermo Fisher Scientific, Darmstadt, Germany
B. licheniformis DSM13	wildtype	DSMZ
B. subtilis ATCC6051	wildtype	ATCC
B. pumilus Jo2	wildtype	BASF
B. licheniformis BL01	*B. licheniformis* Δ*rms*, Δ*pga*	BASF
B. licheniformis SH003	*B. licheniformis* DSM13 Δ*hsdR1*, Δ*hsdR2*	This study
B. subtilis BS01	*B. subtilis* ATCC6051 Δ*amyE*	This study
B. subtilis BS02	*B. subtilis* ATCC6051 Δ*aprE*	This study
B. licheniformis SH003-GFP	*B. licheniformis* SH003 Δ*amyL::PaprE-gfpmut2*	This study

Table S2: Plasmids used in this study

Plasmid	Description	Reference
pDhsdR1	Deletion of restrictase gene *hsdR1* and putative 5-methylcytosine-specific restriction endonuclease *mcrA*	Dutschei et al. 2022
pDhsdR2	Deletion of restrictase gene *hsdR2*	Dutschei et al. 2022
pCB56C	*Bacillus licheniformis* protease expression vector	Wilson et al. 1994
p689_GG	pUC57-based Golden Gate destination (*Bpi*I) & cargo (*Bsa*I) vector	This study
pJOE8999	*E. coli – Bacillus* shuttle plasmid, pUC18 ori, pE194ts ori, *kanR*, P_{vanP}-*lacZ*-sgRNA, P_{manP}-*cas9*	Altenbuchner 2016
pJOE_GG	pJOE8999 with GG cloning module MCS-IIS B1, *mRFP*, MCS-IIS F2	This study
pJOE_amyL	pJOE8999 with repair template for *amyL* deletion, *amyL*-sgRNA	This study
pJOE_amyL_T	pJOE_amyL with terminators T1T2T0 upstream of P_{manP}-*cas9*	This study
pPv4-1_amyL	pJOE_amyL_T with promoter variant Pv4-1 controlling *cas9*	This study
pPv4-2_amyL	pJOE_amyL_T with promoter variant Pv4-2 controlling *cas9*	This study
pPv4-3_amyL	pJOE_amyL_T with promoter variant Pv4-3 controlling *cas9*	This study
pPv4-4_amyL	pJOE_amyL_T with promoter variant Pv4-4 controlling *cas9*	This study
pPv4-5_amyL	pJOE_amyL_T with promoter variant Pv4-5 controlling *cas9*	This study
pPv4-6_amyL	pJOE_amyL_T with promoter variant Pv4-6 controlling *cas9*	This study
pPv4-7_amyL	pJOE_amyL_T with promoter variant Pv4-7 controlling *cas9*	This study
pPv4-8_amyL	pJOE_amyL_T with promoter variant Pv4-8 controlling *cas9*	This study
pPv4-9_amyL	pJOE_amyL_T with promoter variant Pv4-9 controlling *cas9*	This study
pPv8-1_amyL	pJOE_amyL_T with promoter variant Pv8-1 controlling *cas9*	This study
pPv8-2_amyL	pJOE_amyL_T with promoter variant Pv8-2 controlling *cas9*	This study
pPv8-3_amyL	pJOE_amyL_T with promoter variant Pv8-3 controlling *cas9*	This study
pPv8-4_amyL	pJOE_amyL_T with promoter variant Pv8-4 controlling *cas9*	This study
pPv8-5_amyL	pJOE_amyL_T with promoter variant Pv8-5 controlling *cas9*	This study
pPv8-6_amyL	pJOE_amyL_T with promoter variant Pv8-6 controlling *cas9*	This study

pPv8-7_amyL	pJOE_amyL_T with promoter variant Pv8-7 controlling *cas9*	This study
pPv8-8_amyL	pJOE_amyL_T with promoter variant Pv8-8 controlling *cas9*	This study
pPv4-5_GG	pPv4-5_amyL with P_{vanP}-*lacZ*-sgRNA, GG cloning module MCS-IIS B1, *mRFP*, MCS-IIS F2	This study
pPv4-7_GG	pPv4-7_amyL with P_{vanP}-*lacZ*-sgRNA, GG cloning module MCS-IIS B1, *mRFP*, MCS-IIS F2	This study
pPv8-7_GG	pPv8-7_amyL with P_{vanP}-*lacZ*-sgRNA, GG cloning module MCS-IIS B1, *mRFP*, MCS-IIS F2	This study
pPv4-5_hag	pPv4-5_GG with repair template for *hag* deletion, *hag*-sgRNA	This study
pPv4-7_hag	pPv4-7_GG with repair template for *hag* deletion, *hag*-sgRNA	This study
pPv8-7_hag	pPv8-7_GG with repair template for *hag* deletion, *hag*-sgRNA	This study
pPv4-5_degU32	pPv4-5_GG with repair template for *degU32* mutation, *degU*-sgRNA	This study
pPv4-7_degU32	pPv4-7_GG with repair template for *degU32* mutation, *degU*-sgRNA	This study
pPv8-7_degU32	pPv8-7_GG with repair template for *degU32* mutation, *degU*-sgRNA	This study
pPv4-5_amyE	pPv4-5_GG with repair template for *amyE* deletion, *amyE*-sgRNA	This study
pPv4-7_amyE	pPv4-7_GG with repair template for *amyE* deletion, *amyE*-sgRNA	This study
pPv8-7_amyE	pPv8-7_GG with repair template for *amyE* deletion, *amyE*-sgRNA	This study
pPv4-5_aprE	pPv4-5_GG with repair template for *aprE* deletion, *aprE*-sgRNA	This study
pPv4-7_aprE	pPv4-7_GG with repair template for *aprE* deletion, *aprE*-sgRNA	This study
pPv8-7_aprE	pPv8-7_GG with repair template for *aprE* deletion, *aprE*-sgRNA	This study
pPv4-5_gfp	pPv4-5_GG with repair template for *amyl::P_{aprE}-gfpmut2* integration, *amyL*-sgRNA	This study
pPv4-5_sigE	pPv4-5_GG with repair template for *sigE* deletion, *sigE*-sgRNA	This study
pPv4-5_sigF	pPv4-5_GG with repair template for *sigF* deletion, *sigF*-sgRNA	This study
pPv4-5_spoIIE	pPv4-5_GG with repair template for *spoIIE* deletion, *spoIIE*-sgRNA	This study

Table S3: Oligonucleotides used in this study

**Oligonucleotide**	**Sequence (5’ > 3’)**
amyL_SP_1	TACGTCGCAGCAGAAATTAAGAGA
amyL_SP_2	AAACTCTCTTAATTTCTGCTGCGA
amyL_Sc_1	TTGCCCGAATACAACGACAG
amyL_Sc_2	TGCGACCTTCTTTGTGCTTGG
CC_t1t2t0_1	CAAATTTACAAAAGCGACTCGTGAGTTTTCGTTCCACTGAG
CC_t1t2t0_2	GAACGTTGCTCTAGAGTTAAGGGATTTTGGTCATGG
CC_t1t2t0_3	GTGGAACGAAAACTCACGAGTCGCTTTTGTAAATTTGG
CC_t1t2t0_4	CATGACCAAAATCCCTTAACTCTAGAGCAACGTTCTTGCC
CC_Pv	AACACGAGCCCATTTTTGTCAAATAAAATTTAACCGGTATCAACGTTAATAAGACGTTGTCAATAAAATTATTTTGACAAAATTTTAATAATCCAAATGAG
CC_Pv4	CTATTGAGTATTTCTTATCCATGTTTGTCCTCCTTATTAGTTAATCTTTCTCCACATTTATTCTCCAACACGAGCCCATTTTTGTCA
CC_R0_cas9	GATTAACTAATAAGGAGGACAAACATGGATAAGAAATACTCAATAGGC
CC_Pv8	CTATTGAGTATTTCTTATCCATGTTTGTCCTCCTTATTAGTTAATCTTTCTCCACATTTATTCTTCAACACGAGCCCATTTTTGTCA
CC_GG_1	GAAGTTTTAGATGCCACTCTTATCCATCAATCCATCACTG
CC_GG_2	CATCTCAAATTTCGCATTTATTCCAATTTCCTTTTTGCGTG
CC_GG_3	CACACGCAAAAAGGAAATTGGAATAAATGCGAAATTTGAG

CC_GG_4	GACCAGTGATGGATTGATGGATAAGAGTGGCATCTAAAAC
hag_B_fw	CAAGCTATGCTTGCTCAAGC
hag_A_rv	CTGTTGGTTCGCTTGAGCAAGCATAGCTTGGACGGTTCAGCGTGTTAAGC
hag_A_fw	GGTGGTGGTCTCTACCCCGATCGAAGAGCCATTCGAG
hag_B_rv	GGTGGTGGTCTCTTGAGCTTCCTCTGTCCGATTGTCC
hag_SP_1	TACGGCAATCTCTGAAAAAATGAG
hag_SP_2	AAACCTCATTTTTTCAGAGATTGC
degU32_SP_1	TACGGGATTTCGAACCTACCTTTG
degU32_SP_2	AAACCAAAGGTAGGTTCGAAATCC
PaprE_fw	TATATGAAGACCTTCGACCCGGGACCTCTTTCCCTGC
PaprE_rv	TATAGAAGACTTATATCTCACTCTCCTCCTCTTTATTCAGA
amyL_B_rv	TATAGGTCTCAACCCACCTGCGATGACCCGTCTCTACCCGAAGACTTACCCGATATCATAATGCCGTCGCACTGG
amyL_B_fw	CCTTGGTCTCGTCGCACCTGCGGATGTCGCGTCTCGTCGCGAAGACAATCGCGATATCTAGAAGAGCAGAGAGGACGG
amyL_A_rv	TTGAGAGGTCTCAGAGAATTGGATCATTTTCCCTATATTTTCTTCC
amyL_A_fw	GTGAGGTCTCATGAGAAGACAATGAGTCTGAGATATCACCATCCGTTATTGACAAGG
hag_Sc_1	TGATCTTGATGAAACGACGG
hag_Sc_2	TAATCCTGATATTCTGATCGCC
degU_Sc_1	ATGATTTAAGGCCGATGGC
degU_Sc_2	ATCCGCCTTCAGCTACTACTT
amyE_Sc_1	GGTCATGAATAATCTGCGTAATAGAC
amyE_Sc_2	GCGTGTACGTTTTGAGGCGCTGCGCC
aprE_Sc_1	GAGCTGGCAGATGAAGCCAATATTCC
aprE_Sc_2	GTACGCGCATGAGGAACGACAAATAAG
sigE_Sc_1	GAGGAGGCATGATCGGAGTTCATTC
sigE_Sc_2	CTCCTCCGTCATTATAGATCGGTTC
sigF_Sc_1	GTGACGAATTATTTGGAAACAGAGG
sigF_Sc_2	CGACATAATGATCGAGATCGAGCTG
spoIIE_Sc_1	CTCAACAACAACAATCAAGACCGAG
spoIIE_Sc_2	GATAGTGAATCGAATCGAGGCGTCC
16SrRNA_fw	GAGGGTTTCCGCCCTTTAGT
16SrRNA_rv	CCCAGGCGGAGTGCTTAA
kanR_fw	GTTCCACGATGCGATTTGTG
kanR_rv	GCCATTTGCCTGCATATTCA
repF_fw	AGGATCGTCCATGTGAAGCAT
repF_rv	CCGCAGATTGCAGGAAGTG
cas9_fw	CCGCTGTCTCTCCACTGTCA
cas9_rv	ACAGACCGCCACAGTATCAAAA
16SrRNA_T7_fw	TAATACGACTCACTATAGGGGAGGGTTTCCGCCCTTTAGT
kanR_T7_fw	TAATACGACTCACTATAGGGGTTCCACGATGCGATTTGTG
repF_T7_fw	TAATACGACTCACTATAGGGAGGATCGTCCATGTGAAGCAT
cas9_T7_fw	TAATACGACTCACTATAGGGCCGCTGTCTCTCCACTGTCA

Table S4: Nucleotide sequences of gene synthesis fragments

gene synthesis fragment	Description	Sequence (5’ > 3’)
Syn_1	GG cloning module of pBSd141R; MCS-IIS B1, *mRFP*, MCS-IIS F2	GGAATAGATCTGGCCAACGAGGCCTCGAGGATCCGATATCATGCATGGCG<br>CGCCACCCTGAGACGACCCTGATGCAGGTGACCCTGAGACCTTCGCGCCC<br>AGCTGTCTAGGGCGGCGGATTTGTCCTACTCAGGAGAGCGTTCACCGACA<br>AACAACAGATAAAACGAAAGGCCCAGTCTTTCGACTGAGCCTTTCGTTTTAT<br>TTGATGCCTTTAATTAAGTACCTTGTCGGATAAAGCTGTGTTATATTATGTCT<br>TGGTGTTAAATACACACGCTTAACGATTTATGCAGAGGGTGCTGCAGGCGG<br>CAGTTCTGTACAAAAATGACCTAAGCGGAGGAAAAAAACCATTATATTAGGA<br>GGAAATAACATGGCCTCTTCAGAGGATGTTATTAAAGAATTTATGCGGTTTA<br>AGGTGAGGATGGAAGGCTCGGTGAACGGACATGAGTTCGAAATTGAGGGA<br>GAAGGTGAAGGCCGCCCTTATGAAGGTACTCAGACAGCGAAATTGAAAGTC<br>ACGAAAGGCGGACCGCTGCCGTTTGCTTGGGACATTCTCTCACCTCAATTT<br>CAATATGGCTCAAAAGCCTACGTAAAACACCCGGCTGACATCCCTGATTACT<br>TAAAGCTATCCTTCCCGGAGGGCTTTAAATGGGAACGAGTTATGAATTTTGA<br>GGACGGCGGCGTCGTTACTGTCACACAGGATTCTTCCCTTCAGGATGGCG<br>AATTTATTTACAAAGTAAAACTTCGTGGAACTAACTTCCCAAGTGATGGTCC<br>CGTGATGCAAAAAAAAACAATGGGATGGGAAGCATCTACGGAACGTATGTA<br>TCCGGAGGATGGAGCCTTAAAGGGTGAAATCAAAATGCGCCTGAAACTTAA<br>AGATGGCGGACACTATGACGCGGAAGTTAAAACAACATATATGGCTAAAAA<br>ACCAGTCCAACTGCCGGGAGCATATAAGACGGATATAAAGTTGGACATTAC<br>CAGCCATAATGAAGATTACACGATTGTGGAACAGTATGAGAGAGCAGAGGG<br>CAGACATAGCACAGGCGCGTAAGAATTAATGAAAAATAAGCGGCAGCCTGC<br>TTTTCCATGCGGGCTGCCGCTTATCGGGTTATTGTCGTGACTGGGAAAACC<br>CTGGCGACTAGTCTTGGACTCCTGTTGATAGATCCAGTAATGACCTCAGAA<br>CTCCATCTGGATTTGTTCAGAACGCTCGGTTGCCGCCGGGCGTTTTTTATT<br>GGTGAGAATCCAGGGGTCCCCAATAATTACGATTTGGTCTCACTCACACCT<br>GCTCGTCTCACTCAATTTAAATGGCGGCCGCGGATCCTCGACGGGCCAATA<br>AGGCCAGATCTGGATT
Syn_2	*B. licheniformis degU32* homology directed repair (HDR) template	GGTCTCGACCCGATTTGGGATTGATACCGACGCTCAGAAAATACTTGAACA<br>CGATCGAAGATTATCATGGAAAAGCAAAGATTCATTTCCAATGCATCGGAGA<br>ATCCGAAGAAAGAAGAATAGCACCGCGGTTTGAGGTTGCACTATTCCGGCT<br>TGCACAGGAAGCGGTGACAAACGCCTTAAAACACTCCGAATCAACTGAAAT<br>TCATGTTAAAGTAGAAGTGACAAAAGATTTTGTGACGCTGATTATCAAAGAC<br>AATGGAAACGGCTTTGACTTAAAAGAAGTAAAAGGCAAGAAGAACAAATCTT<br>TCGGTCTGCTAGGTATGAAAGAAAGAGTCGATTTGCTCGAAGGCTCAATGA<br>CAATCGATTCGAAAATAGGTCTTGGGACATTTATATTGATTAAAGTTCCACT<br>GTCTTTGTAAAGATAATTGTAAAATAGAGACAAAAGACATATTGACCATAAAA<br>GCGGTGTGTTTAACAATGAGAATGGGGAGGCGTAGCTTGTGACTAAAGTAA<br>ATATTGTAATTATTGACGATCTGCAGTTATTCCGTGAAGGTGTCAAACGGAT<br>TTTGGATTTCGAGCCTACCTTTGAAGTAGTGGCCGAAGGAGACGACGGAGA<br>TGAAGCGGCTCGCATTGTCGAGCACTACCATCCTGATGTTGTTATCATGGA<br>TATTAATATGCCGAATGTGAACGGAGTAGAAGCGACAAAACAACTGGTCGA<br>CTTGTATCCGGAATCAAAGGTTATTATTTTATCCATCCATGATGACGAAAACT<br>ATGTTACACATGCATTAAAAACAGGAGCCCTCAGGAGACC
Syn_3	*B. subtilis amyE* spacer-sgRNA & homology directed repair (HDR) template	GGTTGCGGTCTCATACGTGAAGATCAGGCTATCACTGGTTTTAGAGCTAGA<br>AATAGCAAGTTAAAATAAGGCTAGTCCGTTATCAACTTGAAAAAGTGGCACC<br>GAGTCGGTGCTTTTTACTCCATCTGGATTTGTTCAGAACGCTCGGTTGCCG<br>CCGGGCGTTTTTTATCTAAAGCTTAGGCCCAGTCGAAAGACTGGGCCTTTTT<br>AATACGACTCACTATAGGGTCGACGGCCAACGAGGCCGCGGCATTATGTTT<br>GAATTTCCGTTTAAAGAATGGTCTGCAAGCCTTGTGTTTTTGTTCATCATTAT<br>CTTATATTACTGCATCAGGGCTGCGGCATCCGGAATGCTCATGCCGAGAAT<br>AGACACCAAAGAAGAACTGCAAAAACGGGTGAAGCAGCAGCGAATAGAATC<br>AATTGCTTGCGCCTTTGCGGTAGTGGTGCTTACGATGTACGACAGGGGGAT<br>TCCCCATACATTCTTCGCTTGGCTGAAAATGATTCTTCTTTTTATCGTCTGCG<br>GCGGCGTTCTGTTTCTGCTTCGGTATGTGATTGTGAAGCTGGCTTACAGAA<br>GAGCGGTAAAAGAAGAAATAAAAAAGAAATCATCTTTTTTGTTTGGAAAGCG<br>AGGGAAGCGTTCACAGTTTCGGGCAGCTTTTTTTATAGGAACATTGATTTGT<br>ATTCACTCTGCCAAGTTGTTTTGATAGAGTGATTGTGATAATTTTAAATGTAA<br>GCGTTAACAAAATTCTCCAGTCTTCACATCGGTTTGAAAGGAGGAAGCGGA<br>AGAATGAAGTAAGAGGGATTTTTGACTCCGAAGTAAGTCTTCAAAAAATCAA<br>ATAAGGAGTGTCAAGATGAGGGCAAGGCTAGACGGGACTTACCGAAAGAA<br>ACCATCAATGATGGTTTCTTTTTTGTTCATAAATCAGACAAAACTTTTCTCTT<br>GCAAAAGTTTGTGAAGTGTTGCACAATATAAATGTGAAATACTTCACAAACA<br>AAAAGACATCAAAGAGAAACATACCCTGCAAGGATGATTAATGATGAACAAA<br>CATGTAAATAAAGTAGCTTTAATCGGAGCGGGTTTTGTTGGAAGCAGTTATG<br>CATTTGCGTTAATTAACCAAGGGATCACAGATGAGCTTGTGGTCATTGATGT<br>AAATAAAGAAAAAGCAATGGGCGATGTGATGGATTTACCCCACGGAAAGGC<br>GTTTGGGCTACAACCGGTCAAAACATCTTACGGAACATATGAAGACTGCAA<br>GGATGCTGATATTGTCTGCATTTGCGCCGGAGCAAACCAAAAACCTGGTGA<br>GACACGCCTTGAATTAGTAGAAAAGAACTTGAAGATTTTCAAAGGCATCGTT<br>AGTGAAGTCATGGCGAGCGGATTTGACGGCATTTTCTTAGTCGCGACAAAT<br>CCGGTTGATATCCTGACTTACGCAACATGGAAATTCAGCGGCCTGCCAACT<br>CAAGAGACCCGTTG

Syn_4	*B. subtilis aprE* spacer-sgRNA & homology directed repair (HDR) template	GGTTGCGGTCTCATACGGAAACAAACCCATACCAGGAGTTTTAGAGCTAGA<br>AATAGCAAGTTAAAATAAGGCTAGTCCGTTATCAACTTGAAAAAGTGGCACC<br>GAGTCGGTGCTTTTTACTCCATCTGGATTTGTTCAGAACGCTCGGTTGCCG<br>CCGGGCGTTTTTTATCTAAAGCTTAGGCCCAGTCGAAAGACTGGGCCTTTTT<br>AATACGACTCACTATAGGGTCGACGGCCAACGAGGCCCATTTTCTTCTGCT<br>ATCAAAATAACAGACTCGTGATTTTCCAAACGAGCTTTCAAAAAAGCCTCTG<br>CCCCTTGCAAATCGGATGCCTGTCTATAAAATTCCCGATATTGGTTAAACAG<br>CGGCGCAATGGCGGCCGCATCTGATGTCTTTGCTTGGCGAATGTTCATCTT<br>ATTTCTTCCTCCCTCTCAATAATTTTTTCATTCTATCCCTTTTCTGTAAAGTTT<br>ATTTTTCAGAATACTTTTATCATCATGCTTTGAAAAAATATCACGATAATATCC<br>ATTGTTCTCACGGAAGCACACGCAGGTCATTTGAACGAATTTTTTCGACAGG<br>AATTTGCCGGGACTCAGGAGCATTTAACCTAAAAAAGCATGACATTTCAGCA<br>TAATGAACATTTACTCATGTCTATTTTCGTTCTTTTCTGTATGAAAATAGTTAT<br>TTCGAGTCTCTACGGAAATAGCGAGAGATGATATACCTAAATAGAGATAAAA<br>TCATCTCAAAAAAATGGGTCTACTAAAATATTATTCCATCTATTACAATAAATT<br>CACAGAATAGTCTTTTAAGTAAGTCTACTCTGAATTTTTTTAAAAGGAGAGG<br>GTAAAGATAATAGTAAAAAGAAGCAGGTTCCTCCATACCTGCTTCTTTTTATT<br>TGTCAGCATCCTGATGTTCCGGCGCATTCTCTTCTTTCTCCGCATGTTGAAT<br>CCGTTCCATGATCGACGGATGGCTGCCTCTGAAAATCTTCACAAGCACCGG<br>AGGATCAACCTGGCTCAGCCCCGTCACGGCCAAATCCTGAAACGTTTTAAC<br>AGCGGCTTCTCTGTTCTCTGTCAACTCGATCCCATACTGGTCAGCCTTATTC<br>TCCTGATAACGCGAGACAGCATTAGAAAAAGGCGTAACCGCAAAGCTCAAA<br>ACAGAAAACAAAAGCAATAACAGCGGAAGTGCCGCAAGATCATGCCGCCCT<br>TCTAAATGAAACATGCTGCGGGTTAGGCGAACCGTCCGCTTGTAAAGCTTA<br>TCAATGACATAAAATCCGGCGAGCGACACGAGCAAATAGCCAGCCAGACC<br>GATGTAAACGTGCTTCATGACATAATGGCCCATTTCGTGGCCCATAATAAAC<br>AGAATTTCTGAATCGTCAAGTTTGTTCAGCGTCGTATCCCACAATACAATCC<br>GTTTATTGGCCCCAATTCTCAAGAGACCCGTTG
Syn_5	*B. pumilus spoIIE* spacer-sgRNA & homology directed repair (HDR) template	GGTTGCGGTCTCATACGCTTGTAGCTGAACAGCTGATGTTTTAGAGCTAGA<br>AATAGCAAGTTAAAATAAGGCTAGTCCGTTATCAACTTGAAAAAGTGGCACC<br>GAGTCGGTGCTTTTTACTCCATCTGGATTTGTTCAGAACGCTCGGTTGCCG<br>CCGGGCGTTTTTTATCTAAACTAGTAGGCCCAGTCGATACGTAAAGACTGG<br>GCCTTTTTAATACGACTCACTATAGGGTCGACGGCCAACGAGGCCTCGAGG<br>ATCCGATATCATGCATGGCGCGCCACCCTGAGACGACCCTGATGCAGGTG<br>ACCCGGATCCACTAGTCCTGCTGTCCGCAGGTGCTTTTTTTCTTGACCCAC<br>ACGACATTTTTTGAGATTTCGTCATTTAATTTAAAACTTCCTATTGACGGACA<br>AGCGATTCCTTTGTATTATAGATCTTGTGCTTCTTAGCGCATTTTTATTATGG<br>CGGTGTAGCTCAGCTGGCTAGAGCGTACGGTTCATACCCGTGAGGTCGGG<br>GGTTCGATCCCCTCCGCCGCTATCCTTTTGATTAGAACATAAAAGCAAGGC<br>CCGTTGGTCAAGCGGTTAAGACACCGCCCTTTCACGGCGGTAACACGGGT<br>TCGAATCCCGTACGGGTCATCTTCGAAAACAGCTTTCTTTAGGAAAGCTGTT<br>TTTTTGTGTCTTCATAAAATTCTGATGAAAGACATCGACTTTCAAGAAAGTAT<br>GCCTCTTTGACGAATAAAGCGTCGAACGTTTTATGGAAACGACAACTTCTTT<br>TGACAAAATTTCTTTTTCACCTTCGCTATAATGACAAGCAACGAATATCAGTG<br>AAATATCGTATAATATGAATTTCTTCTGGCGATGATGGGGATATAAAGCATT<br>CAGTACGATCCCAGGAGGAATGAAGATGCGAAAAGGTCACGTAAACCAAAT<br>CTTATTGATTACAGATGGCTGCTCAAATCACGGGGAAGATCCACTTGCGATT<br>GCCTCATTGGCAAAGGAACAAGGGATTACAGTCAATGTTATTGGCATTATG<br>GAGGAAAACAGACACGACCATGAAGCAATGAAAGAAGTTGAAGGGATTGCT<br>CTCGCAGGTGGAGGCATCCATCAAGTTGTCTACGTCCAGCAGTTATCTCAA<br>ACCGTACAAATGGTTACAAAAAAAGCGATGACACAAACCTTGCAAGGTGTT<br>GTGAATAAAGAATTGCAGCAAATACTTGGCAAGGACACTGAAATTGAAGAG<br>CTGCCACCTGATAAACGCGGGGAAGTGATGGAAGTAGTCGATGAGTTAGG<br>AGAGACGGTTCATCTTCAAGTGCTTGTGCTTGTTGATACTCAAGAGACCCGT<br>TG
Syn_6	*B. pumilus sigF* spacer-sgRNA & homology directed repair (HDR) template	GGTTGCGGTCTCATACGTGTCGTCCTCGCTCAAGAAGGTTTTAGAGCTAGA<br>AATAGCAAGTTAAAATAAGGCTAGTCCGTTATCAACTTGAAAAAGTGGCACC<br>GAGTCGGTGCTTTTTACTCCATCTGGATTTGTTCAGAACGCTCGGTTGCCG<br>CCGGGCGTTTTTTATCTAAACTAGTAGGCCCAGTCGATACGTAAAGACTGG<br>GCCTTTTTAATACGACTCACTATAGGGTCGACGGCCAACGAGGCCTCGAGG<br>ATCCGATATCATGCATGGCGCGCCACCCTGAGACGACCCTGATGCAGGTG<br>ACCCGGATCCACTAGTAAATTATCCGTATGGAGCCTTCAGAGCAAACGGCA<br>TTGCAAACATTGGGGGTGGCATCATGAGGAATGAAATGAACCTGACCTTCT<br>CTGCCTTAAGTCAAAATGAATCCTTTGCGAGGGTGACAGTCGCGGCGTTTA<br>TCGCCCAGCTTGATCCGACATTAGATGAATTAACTGAAATCAAAACCGTTGT<br>GTCAGAGGCGGTGACAAACTCCATTATCCATGGCTATGATGGGAATCCAGA<br>TGGCAAGGTGCATATTGAAGTCACACTTGATGATCATGTTGTGTACCTGACC<br>ATCCGTGACGAAGGAATGGGTATTACAGATCTTGAGGAAGCAAGACAGCCG<br>CTTTTCACGACAAAACCAGACTTAGAACGCTCTGGCATGGGCTTTACCATTA<br>TGGAGAATTTTATGGATGATGTCATGATAGACTCATCTCCAGAAATGGGCAC<br>AACCATCCGTTTAACAAAGCATCTATCAAAAAGCAAAGCGCTTTGTAATTAA<br>ATAGCCAATTCGGCTGGCTTTTTTTGTGTGGTAATTACCGGTAAATGAAGTT<br>CTCTCGGTATGAGAACCATTTTTCACCACATACTATTTTTAACCCATCGTATA<br>GGAAGTGACTTGGATGGACGGACAAATCTTTCTTCGGCTGCGCCATCGCAT<br>TAAGACCGGTAATGATCAGCTCATTTATTTAGAAGATATCGCCCAAATCACT<br>GGTGATGAGTTGGCTGTGCAAAAGCTTAGCAAGATGCCGATATATCATGTC<br>AGTAAAAAGGATCGTCACATTGCCGTTCTTGATATCATGCATGTGGTCAAAA<br>CGATCAAAAAAACATGGCCAACCATCGACATTCAAACTGTCGGAGGCGCTG

		AAGCCATTGTTGAAATTGATACAGGCAAACGCCAGCTTTCTCCCGTATTATT<br>TGTGTTCGTGTGGCTTTTATTATTTGTCGGAGCGGCGCTTGCCATTATGAAT<br>TTCCACGAGGATGTCAGTATGCGGCTCGTCCATATCTCAAGAGACCCGTTG
Syn_7	*B. pumilus sigE* spacer-sgRNA & homology directed repair (HDR) template	GGTTGCGGTCTCATACGCGGTGAGGAAAAAACCCAAAGTTTTAGAGCTAGA<br>AATAGCAAGTTAAAATAAGGCTAGTCCGTTATCAACTTGAAAAAGTGGCACC<br>GAGTCGGTGCTTTTTACTCCATCTGGATTTGTTCAGAACGCTCGGTTGCCG<br>CCGGGCGTTTTTTATCTAAACTAGTAGGCCCAGTCGATACGTAAAGACTGG<br>GCCTTTTTAATACGACTCACTATAGGGTCGACGGCCAACGAGGCCTCGAGG<br>ATCCGATATCATGCATGGCGCGCCACCCTGAGACGACCCTGATGCAGGTG<br>ACCCGGATCCACTAGTAAGACAGTATGATGAACAAGTGCTTGTGGAACTAC<br>ACATTCACGGAGAGACAATTCGTTTAAAGGGACTCGTCGATTCTGGCAACC<br>AGCTGTATGATCCTATGACCAAAACACCGGTCATGATCGTCCAGGCCGACC<br>ATCTAACGGCCATTTGCGGAGAATCGTTTATAGACCTTATGAAACAGTCTCA<br>TCCTGTTGAAGTCATGCAAAAGATCGATGATCAATTTCCTCTTCTTGATCGA<br>TTAAGACTTGTTCCATATCGAGCAGTCGGTCATGATCACGGTTTTCTACTAT<br>GCCTAAAACCAGATACAGTTGTCATTTATTCAAAGACGCATATGATTCAGCC<br>AGCTAAGTGTTTTGTAGGATTGAGTCTGAGCGGCTTATCGGCAGATCAGGA<br>ATTTCAATCCATCATTCATCCAGATATGTTAGACGGGAAAATCATCCAGGGG<br>GTGTCGTAGTTTTTGGTGATGTCTTTTATCTTACGAGGTCAACTTGACATTTT<br>GAAAAATTTTTTTGAAAAGCTCTGTCCCTGTACTGTCAAAGGAAACAACCTTT<br>TTTCTCATGATTCTCGTCATCGCTCGTGCATATTTTTCCAACCCAAGGAGAT<br>ACTGAACTTTGTACAACAGCTCCTGTAGGGAGGGAAAAAAGTGTCCAGAAA<br>TAAAGTGGAAATCTGCGGAGTCGACACCTCCAAGCTGCCTGTTCTGAAAAA<br>CGACGAGATGAGAAAATTGTTCAGACAGCTGCAAGATGAGGGTGACGATAC<br>AGCAAGAGAAAAGCTAGTCAATGGCAATTTACGGTTGGTTTTAAGTGTGATT<br>CAGCGTTTCAACAACAGAGGCGAGTATGTCGATGATCTCTTTCAAGTAGGC<br>TGTATCGGATTAATGAAATCAATTGATAATTTTGATTTAAGCCACAATGTCAG<br>ATTTTCAACTTATGCGGTTCCTATGATCATAGGAGAAATCCGTCGATACTTG<br>CGTGATAATAATCCGATTCGGGTGTCTCGCTCACTCAAGAGACCCGTTG
Syn_8	GG compatible GFPmut2 gene variant (genbank accession number AF302837)	ATTAGAAGACCTATATGAGTAAAGGAGAAGAACTTTTCACTGGAGTTGTCCC<br>AATTCTTGTTGAATTAGATGGCGATGTTAATGGGCAAAAATTCTCTGTCAGT<br>GGAGAGGGTGAAGGTGATGCAACATACGGAAAACTTACCCTTAAATTTATTT<br>GCACTACTGGGAAGCTACCTGTTCCATGGCCAACACTTGTCACTACTTTCG<br>CGTATGGTCTTCAATGCTTTGCGAGATACCCAGATCATATGAAACAGCATGA<br>CTTTTTCAAGAGTGCCATGCCCGAAGGTTATGTACAGGAAAGAACTATATTT<br>TACAAAGATGACGGGAACTACAAGACACGTGCTGAAGTCAAGTTTGAAGGT<br>GATACCCTTGTTAATAGAATCGAGTTAAAAGGTATTGATTTTAAAGAAGATG<br>GAAACATTCTTGGACACAAAATGGAATACAACTATAACTCACATAATGTATAC<br>ATCATGGCAGACAAACCAAAGAATGGAATCAAAGTTAACTTCAAAATTAGAC<br>ACAACATTAAAGATGGAAGCGTTCAATTAGCAGACCATTATCAACAAAATAC<br>TCCAATTGGCGATGGCCCTGTCCTTTTACCAGACAACCATTACCTGTCCACA<br>CAATCTGCCCTTTCCAAAGATCCCAACGAAAAGAGAGATCACATGATCCTTC<br>TTGAGTTTGTAACAGCTGCTGGGATTACACATGGCATGGATGAACTATACAA<br>ATAATAGCTTGTCTTCATTA
Syn_9	GG cloning module of p689_GG; MCS-IIS E1, *Bpi*I-*lacZ*-*Bpi*I, MCS-IIS C2	GCGATGTTAAGGACCGTCTCATCTCACCTGCAAGGTCTCATCTCTTTTACTC<br>CATCTGGATTTGTTCAGAACGCTCGGTTGCCGCCGGGCGTTTTTTATCTAAA<br>ACTAGTGTCGAGGGTCTTCGGTACCGCGATTTACATATGCTGGCACGACAG<br>GTTTCCCGACTGGAAAGCGGGCAGTGAGCGCAACGCAATTAATGTGAGTTA<br>GCTCACTCATTAGGCACCCCAGGCTTTACACTTTATGCTTCCGGCTCGTATG<br>TTGTGTGGAATTGTGAGCGGATAACAATTTCACACAGGAAACAGCTATGAC<br>CATGATTACGCCAAGCTTGCATGCCTGCAGGTCGACTCTAGAGGATCCCCG<br>GGTACCGAGCTCGAATTCACTGGCCGTCGTTTTACAACGTCGTGACTGGGA<br>AAACCCTGGCGTTACCCAACTTAATCGCCTTGCAGCACATCCCCCTTTCGC<br>CAGCTGGCGTAATAGCGAAGAGGCCCGCACCGATCGCCCTTCCCAACAGT<br>TGCGCAGCCTGAATGGCGAATGGCGCCTGATGCGGTATTTTCTCCTTACGC<br>ATCTGTGCGGTATTTCACACCGCATATGGTGCACTCTCAGTACAATCTGCTC<br>TGATGCCGCATAGTTAAGCCAGCCCCGACACCCGCCAACACCCGCTGCCG<br>CGTTTATAATGAAGACCCTAGCCGGCGCGCCACAAATAGCATATAGAAAAA<br>GCTAGTGTTTTTAGCACTAGCTTTTTCTTCATTCTGATGAAGGAATAAATTAA<br>TTAAAGGCATCAAATAAAACGAAAGGCTCAGTCGAAAGACTGGGCCTTTCG<br>TTTTATCTGTTGTTTGTCGGTGAACGCTCTCCTGAGTAGGACAAATCCGCCG<br>CCCTAGACAGCTGTCGCTGAGACGATCGCTGAGACCTCGCAAGTTCTCGC<br>CATCGCAGGTGAAATCTAGATGTATTCGC

Table S5: Nucleotide sequences of mutated Pv4 and Pv8 promoter variants from pJOE_amyL_T derived plasmids pPv4-1_amyL - pPv4-9_amyL (Pv4 promoter variants) and pPv8-1_amyL – pPv8-8_amyL (Pv8 promoter variants). In plasmid pPv8-6_amyL the native Pv8 promoter is present, but a frameshift mutation within the *cas9* coding region leads to premature translational stop and a non-active Cas9 (*).

**Plasmid**	**Pv4 / Pv8 variant**	**Sequence (5' > 3')**
pPv4-1_amyL	Pv4-1	AATTTTGTCAAAATAATTTTATTGACAACGTCTTATTAACGTTGATACCGGTTAAATTTTATTTGACAAAATGGGCTCGTGTTGGAGAATAAATGTGGAGAAAGATTAACTAATAAGGAGGACAAAC
pPv4-2_amyL	Pv4-2	AATTTTGTCAAAATAATTTTATTGACAACGTCTTATTAACGTTGATACCGGTTAAATTTTATTTGACAAAAATGGGCTCGTGTTGGAGAAAATGTGGAGAAAGATTAACTAATAAGGAGGACAAAC
pPv4-3_amyL	Pv4-3	AATTTTGTCAAAATAATTTTATTGACAACGTCTTATTAACGTTGATACCGGTTAAATTTTATTTGACAAAAATGGGCTCGTGTTGGGAATAAATGTGGAGAAAGATTAACTAATAAGGAGGACAAAC
pPv4-4_amyL	Pv4-4	AATTTTGTCAAAATAATTTTATTGACAACGTCTTATTAACGTTGATACCGGTTAAATTTTATTTGACAAAAATGGGCTCGTGTTGGGAATAAATGTGGAGAAAGATTAACTAATAAGGAGGACAAAC
pPv4-5_amyL	Pv4-5	AATTTTGTCAAAATAATTTTATTGACAACGTCTTATTAACGTTGATACCGGTTAAATTTTATTTGAACAAAAATGGGCTCGTGTTGGAGAATAAATGTGGAGAAAGATTAACTAATAAGGAGGACAAAC
pPv4-6_amyL	Pv4-6	AATTTTGTCAAAATAATTTTATTGACAACGTCTTATTAACGTTGATACCGGTTAAATTTTATTTGACAAAAATGGGCTCGTGTTGAGAATAAATGTGGAGAAAGATTAACTAATAAGGAGGACAAAC
pPv4-7_amyL	Pv4-7	AATTTTGTCAAAATAATTTTATTGACAACGTCTTATTAACGTTGATACCGGTTAAATTTTATTTGACAAAAATGGGCTCGTGTTGGAAATAAATGTGGAGAAAGATTAACTAATAAGGAGGACAAAC
pPv4-8_amyL	Pv4-8	AATTTTGTCAAAATAATTTTATTGACAACGTCTTATTAACGTTGATACCGGTTAAATTTTATTTGACAAAAAGGGCTCGTGTTGGAGAATAAATGTGGAGAAAGATTAACTAATAAGGAGGACAAAC
pPv4-9_amyL	Pv4-9	AATTTTGTCAAAATAATTTTATTGACAACGTCTTATTAACGTTGATACCGGTTAAATTTTATTTGACAAAAATGGGCTCGTGTTGGAAATAAATGTGGAGAAAGATTAACTAATAAGGAGGACAAAC
pPv8-1_amyL	Pv8-1	AATTTTGTCAAAATAATTTTATTGACAACGTCTTATTAACGTTGATACCGGTTAAATTTTATTTGACAAAAATGGGCTCGTGTTGAAGAAAAATGTGGAGAAAGATTAACTAATAAGGAGGACAAAC
pPv8-2_amyL	Pv8-2	AATTTTGTCAAAATAATTTTATTGACAACGTCTTATTAACCGTTGATACCGGTTAAATTTTATTTGACAAAAATGGGCTCGTGTTGAAGAATAAATGTGGAGAAAGATTAACTAATAAGGGAGGACAAAC
pPv8-3_amyL	Pv8-3	AATTTTGTCAAAATAATTTTATTGACAACGTCTTATTAACGTTGATACCGGTTAAATTTTATTTGACAAAAATGGGCTCGTGTTGAAGAATAAATGTGGAAAAGATTAACTAATAAGGAGGACAAAC
pPv8-4_amyL	Pv8-4	AATTTTGTCAAAATAATTTTATTGACAACGTCTTATTAACGTTGATACCCGGTTAAATTTTATTTGACAAAAATGGGCTCGTGTGAAGAATAAATGTGGAGAAAGATTAACTAATAAGGAGGACAAAC
pPv8-5_amyL	Pv8-5	AATTTTGTCAAAATAATTTTATTGACAACGTCTTATTAACGTTGATACCGGTTAAATTTTATTTGACAAAAATGGGCTCGTGTTGAGAATAAATGTGGAGAAAGATTAACTAATAAGGAGGACAAAC
pPv8-6_amyL*	Pv8	AATTTTGTCAAAATAATTTTATTGACAACGTCTTATTAACGTTGATACCGGTTAAATTTTATTTGACAAAAATGGGCTCGTGTTGAAGAATAAATGTGGAGAAAGATTAACTAATAAGGAGGACAAAC
pPv8-7_amyL	Pv8-7	AATTTTGTCAAAATAATTTTATTGACAACGTCTTATTAACGTTGATACCGGTTAAATTTTATTTGACAAAATGGGCTCGTGTTGAAGAATAAATGTGGAGAAAGATTAACTAATAAGGAGGACAAAC
pPv8-8_amyL	Pv8-8	AATTTTGTCAAAATAATTTTATTGACAACGTCTTATTAACGTTGATACCGGTTAAATTTTATTTTGACAAAAATGGGCTCGTGTTGAAGAATAAATGTGGAGAAAGATTAACTAATAAGGAGGACAAAC

References

Altenbuchner J (2016) Editing of the Bacillus subtilis Genome by the CRISPR-Cas9 System. Appl Environ Microbiol 82:5421–5427. https://doi.org/10.1128/AEM.01453-16

Dutschei T, Zühlke M-K, Welsch N, Eisenack T, Hilkmann M, Krull J, Stühle C, Brott S, Dürwald A, Reisky L, Hehemann J-H, Becher D, Schweder T, Bornscheuer UT (2022) Metabolic engineering enables Bacillus licheniformis to grow on the marine polysaccharide ulvan. Microb Cell Factories 21:207. https://doi.org/10.1186/s12934-022-01931-0

LaFleur TL, Hossain A, Salis HM (2022) Automated model-predictive design of synthetic promoters to control transcriptional profiles in bacteria. Nat Commun 13:5159. https://doi.org/10.1038/s41467-022-32829-5

Wilson CR, Landin BF, Mielenz JR, Hom SSM, Hansen D, Reynolds RB, Kennedy NCT, Schindler J, Bahn M, Schmid R, Markgraf M, Paech C, Maurer K-H (1994) Alkaline proteolytic enzyme and method of production (Patent US5352604A)
